# ﻿Botryosphaerialean fungi associated with woody oil plants cultivated in Sichuan Province, China

**DOI:** 10.3897/mycokeys.97.103118

**Published:** 2023-05-23

**Authors:** Wen-Li Li, Rui-Ru Liang, Asha J. Dissanayake, Jian-Kui Liu

**Affiliations:** 1 School of Life Science and Technology, Center for Informational Biology, Electronic Science and Technology University, Chengdu 611731, China Electronic Science and Technology University Chengdu China

**Keywords:** Botryosphaeriales, diversity, new species, phylogeny, taxonomy

## Abstract

Woody oil plants are important economic trees which are widely cultivated and distributed throughout China. Surveys conducted during 2020 and 2021 on several woody oil plantations from five regions of Sichuan Province, China, revealed a high diversity of Botryosphaerialean fungi. The identification of 50 botryosphaeriaceous isolates was carried out based on both morphology and multi-gene phylogenetic analysis of internal transcribed spacer region (ITS), translation elongation factor 1-alpha gene (*tef1*) and β-tubulin gene (*tub2*). This allowed the identification of twelve previously known Botryosphaeriales species: *Aplosporellaprunicola*, *A.ginkgonis*, *Barriopsistectonae*, *Botryosphaeriadothidea*, *Bo.fabicerciana*, *Diplodiamutila*, *Di.seriata*, *Dothiorellasarmentorum*, *Neofusicoccumparvum*, *Sardiniellaguizhouensis*, *Sphaeropsiscitrigena*, and *Sp.guizhouensis*, and four novel species belonging to the genera *Diplodia* and *Dothiorella*, viz. *Di.acerigena*, *Di.pistaciicola*, *Do.camelliae* and *Do.zanthoxyli*. The dominant species isolated across the surveyed regions were *Botryosphaeriadothidea*, *Sardiniellaguizhouensis* and *Diplodiamutila*, representing 20%, 14% and 12% of the total isolates, respectively. In addition, most isolates were obtained from *Pistaciachinensis* (14 isolates), followed by *Camelliaoleifera* (10 isolates). The present study enhances the understanding of Botryosphaeriales species diversity on woody oil plants in Sichuan Province, China.

## ﻿Introduction

Botryosphaeriaceae is a diverse group of fungi that includes endophytes, saprobes and plant pathogens (Phillips et al. 2013). They have broad host ranges, and are widely distributed in tropical and temperate regions (Batista et al. 2021). Botryosphaeriaceae was introduced by Theissen and Sydow (1918) to accommodate three genera *Botryosphaeria*, *Dibotryon* and *Phaeobotryon*. Botryosphaeriales was proposed to include the single family, Botryosphaeriaceae, based on multi-gene phylogeny (Schoch et al. 2006). Up to date, six families and 32 genera are accepted in Botryosphaeriales, while Botryosphaeriaceae is known to be the largest monophyletic family, including 22 genera and more than 200 species (https://botryosphaeriales.org/, accessed on 15^th^ April 2023).

The members of Botryosphaeriaceae have been taxonomically characterized based on both sexual and asexual morphs. The production of large, ovoid to oblong, typically hyaline, aseptate ascospores, which may become brown and septate with age, as well as bitunicate asci within unilocular or multilocular botryose ascomata known as pseudothecia is typical to the sexual state (Sivanesan 1984; Phillips et al. 2005). The asexual states of Botryosphaeriaceae exhibit a wide range of conidial morphologies; for example, its conidia can be thin-walled and hyaline, or thick-walled and pigmented, aseptate or 1–2-septate (Phillips et al. 2005). Additonaly, the spermatial states were also frequently observed in Botryosphaeriaceae species, which produced unicellular, hyaline, allantoid to rod-shaped spermatia on culture. Botryosphaeriaceae species are significantly different from other fungi in that the color of its aerial hyphae, changing from gray to black with age on 2% potato dextrose agar (PDA), which can be used for the rapid determination of botryosphaeriaceous fungi.

The geographic distribution and host range of botryosphaeriaceous taxa are diverse. Seven genera in Botryosphaeriaceae: *Botryosphaeria*, *Diplodia*, *Dothiorella*, *Lasiodiplodia*, *Neodeightonia*, *Neofusicoccum* and *Phaeobotryon* are common and frequently reported from various geographical regions (Batista et al. 2021), while *Botryobambusa*, *Oblongocollomyces*, *Sakireeta* and *Sardiniella* appear to be limited to a single region or country (Liu et al. 2012; Crous et al. 2015; Linaldeddu et al. 2016; Yang et al. 2017; Dissanayake et al. 2021). Many Botryosphaeriaceae species have wide host ranges (e. g. *Botryosphaeriadothidea*, *Diplodiamutila*, *Dothiorellasarmentorum*, *Lasiodiplodiatheobromae* and *Neofusicoccumparvum*), while other species have narrower host ranges (e. g. *Diplodiaolivarum* was reported on olive, oleaster, carob, grapevine, almond et al.) (Lazzizera et al. 2008; Granata et al. 2011; Linaldeddu et al. 2015; Olmo et al. 2016) or even in very specific hosts (e. g. *Eutiarosporelladarliae* was only reported on infected wheat and wheat-stubble) (Thynne et al. 2015; Farr and Rossman 2021). Different species of Botryosphaeriaceae exhibit different environmental adaptations and host preferences (Braunsdorf et al. 2016). Botryosphaeriaceous taxa with narrow host ranges or limited geographic distribution will be more susceptible to climatic effects (Slippers et al. 2017; Li et al. 2020).

Woody oil plants are economically important as they are used for the production of cooking and industrial oil. Recently, many Botryosphaeriaceae species have been frequently reported on woody oil plants. *Diplodiaolivarum* was first reported from rotting olive drupes in Italy (Lazzizera et al. 2008) and later it was reported as associated with declining *Prunusdulcis* trees in Spain (Gramaje et al. 2012). *Diplodiainsularis* was isolated from branch canker of *Pistacialentiscus* in Italy (Linaldeddu et al. 2015). *Dothiorellagregaria* was isolated from the stems with asymptomatic of *Zanthoxylumbungeanum* in China (Li et al. 2016b). *Botryosphaeriadothidea*, *Diplodiamutila*, *Di.seriata*, *Dothiorellaiberica*, *Do.omnivora*, *Do.sarmentorum*, *Lasiodiplodiacitricola*, *L.pseudotheobromae*, *L.theobromae*, *Neofusicoccummediterraneum*, *N.nonquaesitum*, *N.parvum*, *N.ribis*, *N.vitifusiforme*, and *Neoscytalidiumdimidiatum* have been reported as pathogens of *Englishwalnut* (Juglans regia L.) in California (Chen et al. 2014), Chile (Jimenez Luna et al. 2022), China (Li et al. 2016a; Zhang et al. 2017), Iran (Abdollahzadeh et al. 2013; Panahandeh et al. 2019), South Africa (Cloete et al. 2011), Spain (Gramaje et al. 2012) and USA (Chen et al. 2014). However, very little is known about the Botryosphaerialean species occurring on native woody oil plants in China. Hence, the aim of this study was to gain a more comprehensive understanding of the diversity of Botryosphaeriaceae species associated with common woody oil plants grown in Sichuan Province, China.

## ﻿Materials and methods

### ﻿Isolates and morphology

The isolates in this study were collected from the woody oil tree plantations in Sichuan Province during the period of 2020 and 2021. The hosts include *Acertruncatum*, *Camelliaoleifera*, *Idesiapolycarpa*, *Oleaeuropaea*, *Paeoniasuffruticosa*, *Pistaciachinensis*, *Verniciafordii* and *Zanthoxylumbungeanum*. The samples were collected from decayed stems, branches and twigs of woody oil trees. Mature fruiting bodies were selected for fungal isolation and for morphological observations under stereo microscope Motic SMZ 168 series. Measurements were made with Tarosoft Image Frame Work program v. 0.9.7 (Liu et al. 2010). Thirty conidia/ascospores were measured per isolate, and 10–30 measurements were taken of other morphological structures. At least 20 conidia/ascospores were used to calculate the average length/width ratio (L/W). Single spore isolation was conducted in accordance with the methods described in Chomnunti et al. (2014). Germinated spores were individually placed on PDA plates and grown at 25 °C in daylight.

Herbarium specimens were stored in the herbarium of Cryptogams Kunming Institute of Botany, Academia Sinica (KUN-HKAS) and duplicated at Herbarium, University of Electronic Science and Technology (**HUEST**), Chengdu, China. Living cultures were deposited at China General Microbiological Culture Collection Centre (**CGMCC**), Beijing, China and duplicated at the University of Electronic Science and Technology Culture Collection (**UESTCC**), Chengdu, China. MycoBank numbers were registered as outlined in MycoBank (http://www.MycoBank.org. Accessed on 11^th^ November 2022).

### ﻿DNA extraction, PCR amplification and sequencing

The total genomic DNA was extracted from 7day-old isolates grown on 2% PDA median at 25 °C, using the EZ geneTM fungal gDNA kit (GD2416), following the manufacturer’s instructions and protocols. Partial gene sequences were determined for the internal transcribed spacer 1 and 2 including the intervening 5.8S nrDNA gene (ITS), the nuclear ribosomal 28s large subunit (LSU), the translation elongation factor 1-alphagene (*tef1*), and the β-tubulin gene (*tub2*). The primers used for ampliﬁcation are ITS5/ITS4 for ITS (White et al. 1990), LR0R/LR5 for LSU (Vilgalys and Hester 1990), EF1-728F/EF1-986R for *tef1* (Carbone and Kohn 1999) and Bt2a/Bt2b for *tub2* (Glass and Donaldson 1995). Polymerase chain reaction (PCR) amplification conditions were followed as of Dissanayake et al. (2021). PCR products were sent for sequencing at Beijing Tsingke Biological Engineering Technology and Services Co. Ltd. (Beijing, P.R. China). All newly generated sequences are deposited in GenBank, and the obtained accession numbers are listed in Table 1.

**Table 1.** All newly generated sequences in this study. Ex-type strains are indicated with *. N/A: Not available.

Taxon	Stain Number	GenBank Accession Number		
		ITS	*tef1*	*tub2*
*Aplosporellaginkgonis*	UESTCC 22.0091	OQ190504	OQ241438	N/A
*Aplosporellaprunicola*	UESTCC 22.0090	OQ190505	N/A	N/A
*Barriopsistectonae*	UESTCC 22.0092	OQ190506	OQ241439	N/A
*Botryosphaeriadothidea*	UESTCC 22.0111	OQ190507	OQ241440	N/A
	UESTCC 22.0109	N/A	OQ241441	N/A
	UESTCC 22.0112	OQ190508	OQ241442	N/A
	UESTCC 22.0113	OQ190509	OQ241443	N/A
	UESTCC 22.0108	OQ190510	OQ241444	N/A
	UESTCC 22.0116	OQ190511	OQ241445	N/A
	UESTCC 22.0114	OQ190512	OQ241446	N/A
	UESTCC 22.0115	OQ190513	OQ241447	N/A
	UESTCC 22.0110	OQ190514	OQ241448	N/A
	UESTCC 22.0107	OQ190515	OQ241449	N/A
*Botryosphaeriafabicerciana*	UESTCC 22.0117	OQ190516	OQ241450	N/A
	UESTCC 22.0118	OQ190517	OQ241451	N/A
Diplodiaacerigena*	CGMCC 3.24157	OQ190518	OQ241452	N/A
*Diplodiaacerigena*	UESTCC 22.0074	OQ190519	OQ241453	OQ338163
	UESTCC 22.0075	OQ190520	OQ241454	OQ338164
*Diplodiamutila*	UESTCC 22.0064	OQ190521	OQ241455	OQ338165
	UESTCC 22.0065	OQ190522	OQ241456	OQ338166
	UESTCC 22.0069	OQ190523	OQ241457	OQ338167
	UESTCC 22.0068	OQ190524	OQ241458	OQ338168
	UESTCC 22.0067	OQ190525	OQ241459	OQ338169
	UESTCC 22.0063	OQ190526	OQ241460	OQ338170
*Diplodiapistaciicola* *	CGMCC 3.24156	OQ190527	OQ241461	OQ338171
*Diplodiapistaciicola*	UESTCC 22.0071	OQ190528	OQ241462	OQ275062
*Diplodiaseriata*	UESTCC 22.0072	OQ190529	OQ241463	N/A
*Dothiorellacamelliae*	UESTCC 22.0080	OQ190530	N/A	OQ275063
*Dothiorellacamelliae* *	CGMCC 3.24158	OQ190531	OQ241464	OQ275064
*Dothiorellacamelliae*	UESTCC 22.0079	OQ190532	OQ241465	OQ275065
	UESTCC 22.0078	OQ190533	OQ241466	OQ275066
*Dothiorellasarmentorum*	UESTCC 22.0076	OQ190534	N/A	OQ275067
	UESTCC 22.0077	OQ190535	OQ241467	OQ275068
*Dothiorellazanthoxyli* *	CGMCC 3.24159	OQ190536	OQ241468	OQ275069
*Dothiorellazanthoxyli*	UESTCC 22.0083	OQ190537	OQ241469	OQ275070
	UESTCC 22.0084	OQ190538	OQ241470	OQ275071
*Neofusicoccumparvum*	UESTCC 22.0096	OQ190539	OQ241471	N/A
	UESTCC 22.0094	OQ190540	N/A	N/A
	UESTCC 22.0093	OQ190541	N/A	N/A
	UESTCC 22.0095	OQ190542	N/A	N/A
*Sardiniellaguizhouensis*	UESTCC 22.0100	OQ190543	OQ241472	N/A
	UESTCC 22.0101	OQ190544	OQ241473	N/A
	UESTCC 22.0099	OQ190545	OQ241474	N/A
	UESTCC 22.0097	OQ190546	OQ241475	N/A
	UESTCC 22.0098	OQ190547	OQ241476	N/A
	UESTCC 22.0102	OQ190548	OQ241477	N/A
	UESTCC 22.0103	OQ190549	OQ241478	N/A
*Sphaeropsiscitrigena*	UESTCC 22.0106	OQ190550	OQ241479	N/A
*Sphaeropsiscitrigena*	UESTCC 22.0105	OQ190551	OQ241480	N/A
*Sphaeropsisguizhouensis*	UESTCC 22.0104	OQ190552	OQ241481	N/A

### ﻿Phylogenetic analyses

Sequence data for phylogenetic analyses were obtained from GenBank and from recent publications regarding Botryosphaeriaceae fungi (Dissanayake et al. 2021; Xiao et al. 2021; Zhang et al. 2021; Rathnayaka et al. 2022) (See Suppl. material 1). The single gene alignments were performed using MAFFT v.7.429 online service (https://mafft.cbrc.jp/alignment/server/, accessed on 15 October 2022) (Katoh et al. 2019) and ambiguous regions were excluded using TrimAI with the option “-automated1”, which trimmed sequences based on similarity statistics (Capella-Gutiérrez et al. 2009). Multi-gene sequences were concatenated by Sequence matrix software (Vaidya et al. 2011). Multi-gene phylogenetic analyses were obtained from maximum likelihood (ML) and Bayesian inference (BI) analyses following Dissanayake et al. (2020).

ML analyses was performed using RAxML (Stamatakis 2006). The tree search included 1,000 non-parametric bootstrap replicates and the best scoring tree was selected from suboptimal trees under the GTRGAMMA substitution model. Maximum likelihood bootstrap values equal or greater than 75% are marked near each node of the phylogenetic tree.

Bayesian analyses was performed in MrBayes 3.2.6 (Ronquist et al. 2012). The program MrModeltest 2.2 (Nylander 2004) was used to determine the best nucleotide substitution model for each data partition. The Markov Chain Monte Carlo (MCMC) sampling approach was used to calculate the posterior probabilities (PP) (Rannala and Yang 1996). Bayesian analyses of four simultaneous Markov chains were run for 10,000,000 generations with trees sampled every 1,000^th^ generations. The first 20% of trees, representing the burn-in phase of the analyses, were discarded, and the remaining trees were used for calculating posterior probabilities (PP) in the majority rule consensus tree. PP values equal or greater than 0.95 are marked near each node.

Trees were visualized with FigTree v1.4.0 (Rambaut 2006), and the layout was edited using Adobe Illustrator CS6 software (Adobe Systems, USA).

## ﻿Results

### ﻿Phylogenetic analyses

A concatenated dataset of ITS and *tef1* was used to determine the phylogenetic position of Aplosporellaceae and Botryosphaeriaceae isolates obtained in this study. Combined sequences of ITS and *tef1* were used for the analyses of *Botryosphaeria*, while ITS, *tef1* and *tub2* were used for the analyses of *Diplodia* and *Dothiorella* isolates. All details of the alignments are provided in Table 2.

**Table 2.** Alignment details and ML, BI analyses results of each phylogenetic tree constructed in this study.

Character		Overview phylogenetic tree	*Botryosphaeria*	*Diplodia*	*Dothiorella*
Number of base pairs in each gene region (including the gaps after alignment)		ITS (603 bp), *tef1* (320 bp)	ITS (555 bp), *tef1* (315 bp)	ITS (537 bp), *tef1* (311 bp), *tub2* (381 bp)	ITS (523 bp), *tef 1* (294 bp), *tub2* (427 bp)
Number of isolates obtained in this study		17	12	11	9
Number of taxa originated from GenBank		94	45	64	73
Outgroup taxa		*Lecanostictaacicula* (LNPV252)	*Barriopsisiraniana* (IRAN1448C) and *Barriopsisiraniana* (IRAN1449C)	*Dothiorelladulcispinae* (CMW 36460) and *Dothiorelladulcispinae* (CMW 36462)	*Neofusicoccumluteum* (CBS 562.92) and *Neofusicoccumluteum* (CMW 41365)
BI (model of each gene region)	ITS	GTR+I+G	SYM	K80+I+G	HKY+I+G
	*tef1*	GTR+I+G	HKY+G	GTR+G	GTR+G
	*tub2*	–	–	GTR+G	GTR+I+G

**Figure 1.**
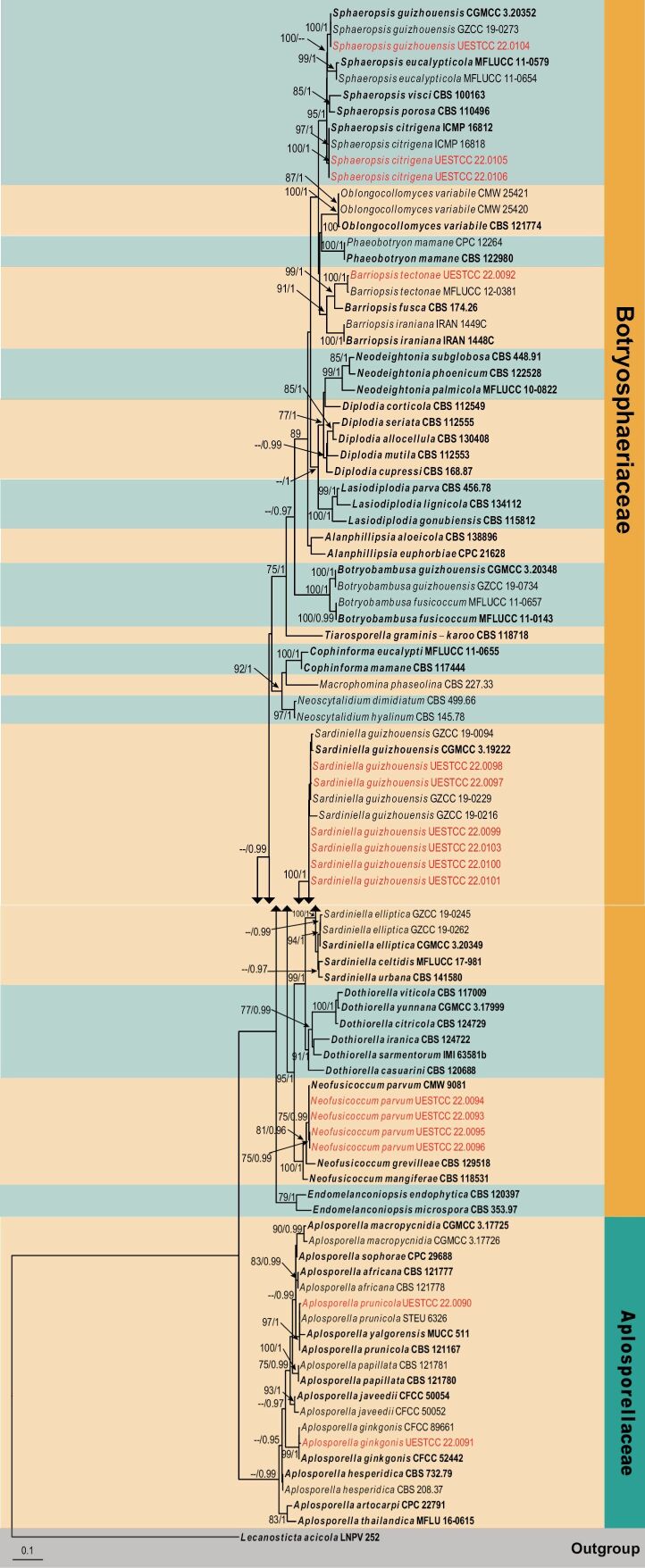
Phylogram generated from RAxML analysis based on combined ITS and *tef1* sequence data of *Botryosphaeriaceae* and *Aplosporellaceae* isolates. The tree was rooted to *Lecanostictaacicula* (LNPV 252). The ML (≥ 75%) and BI (≥ 95%) bootstrap supports are given near the nodes, respectively. Isolates from this study are marked in red and ex-type strains are marked in bold.

In an overview phylogenetic tree (Fig. 1), sixteen newly obtained isolates were nested with four genera of Botryosphaeriaceae, representing seven known species viz. *Barriopsistectonae*, *Neofusicoccumparvum*, *Sardiniellaguizhouensis*, *Sphaeropsisguizhouensis* and *Sp.citrigena*. Two isolates were clustered with the genus *Aplosporella* (Aplosporellaceae), and were identified as *A.ginkgonis* and *A.prunicola*.

**Figure 2.**
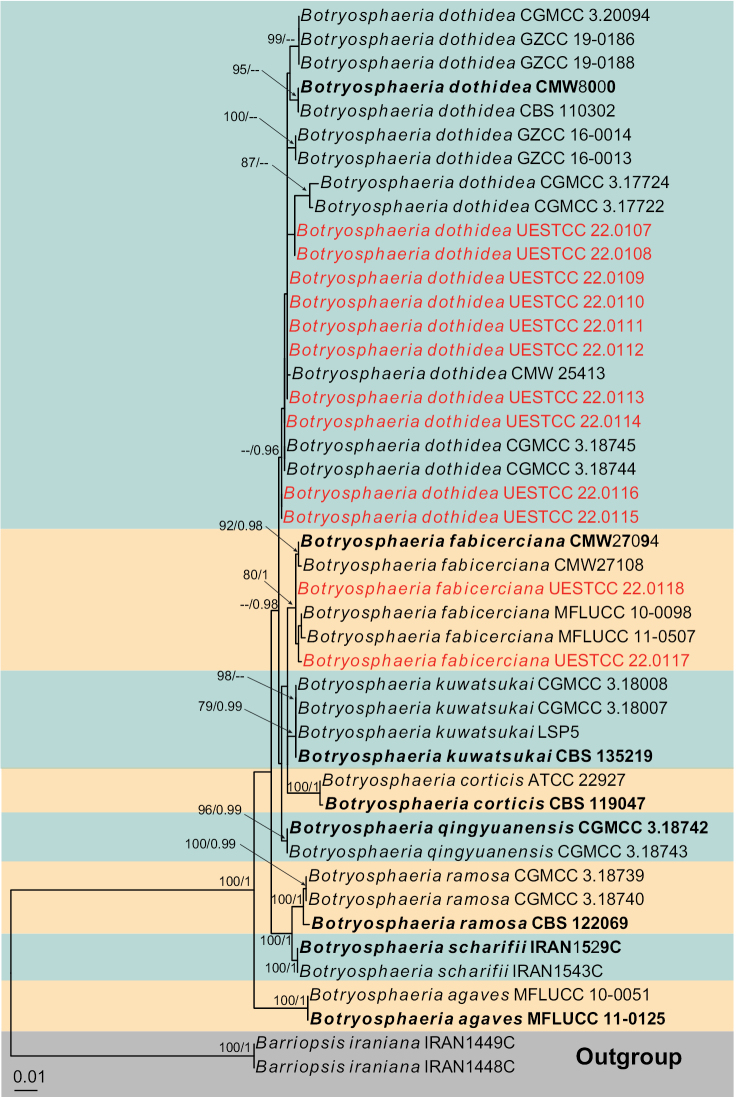
Phylogram generated from RAxML analysis based on combined ITS and *tef1* sequence data of *Botryosphaeria* isolates. The tree was rooted to *Barriopsisiraniana* (IRAN1448C and IRAN1449C). The ML (≥ 75%) and BI (≥ 95%) bootstrap supports are given near the nodes, respectively. Isolates from this study are marked in red and ex-type strains are marked in bold.

**Figure 3.**
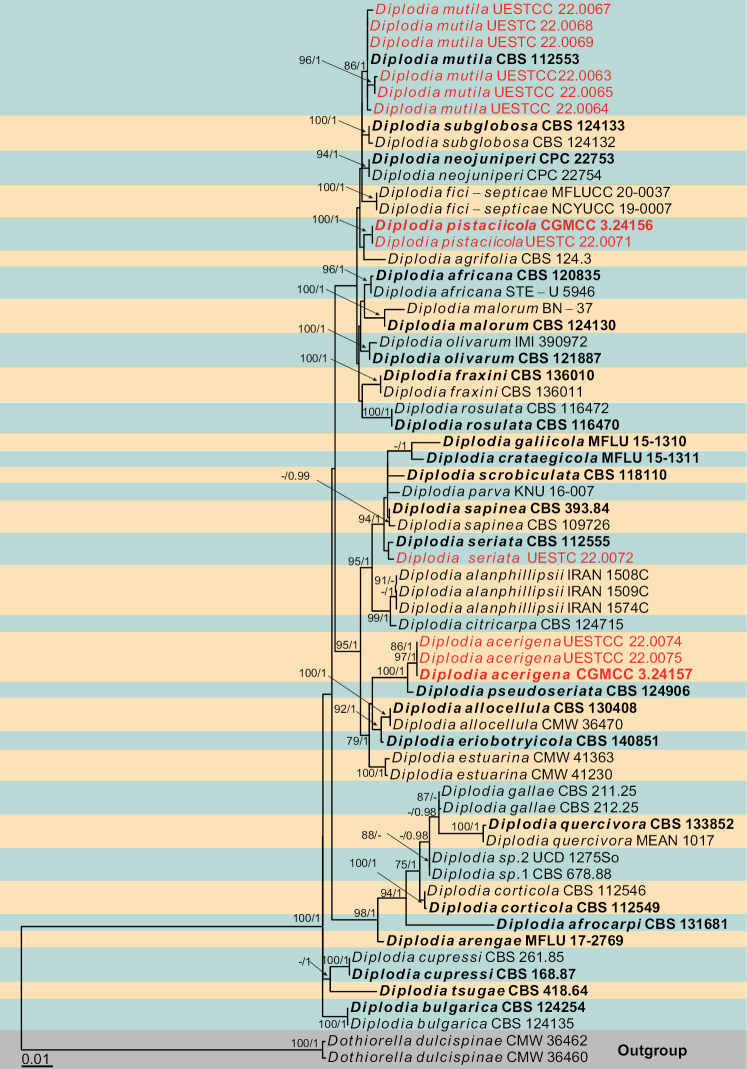
Phylogram generated from RAxML analysis based on combined ITS, *tef1* and *tub2* sequence data of *Diplodia* isolates. The tree was rooted to *Dothiorelladulcispinae* (CMW 36460 and CMW 36462). The ML (≥ 75%) and BI (≥ 95%) bootstrap supports are given near the nodes, respectively. Isolates from this study are marked in red and ex-type strains are marked in bold.

**Figure 4.**
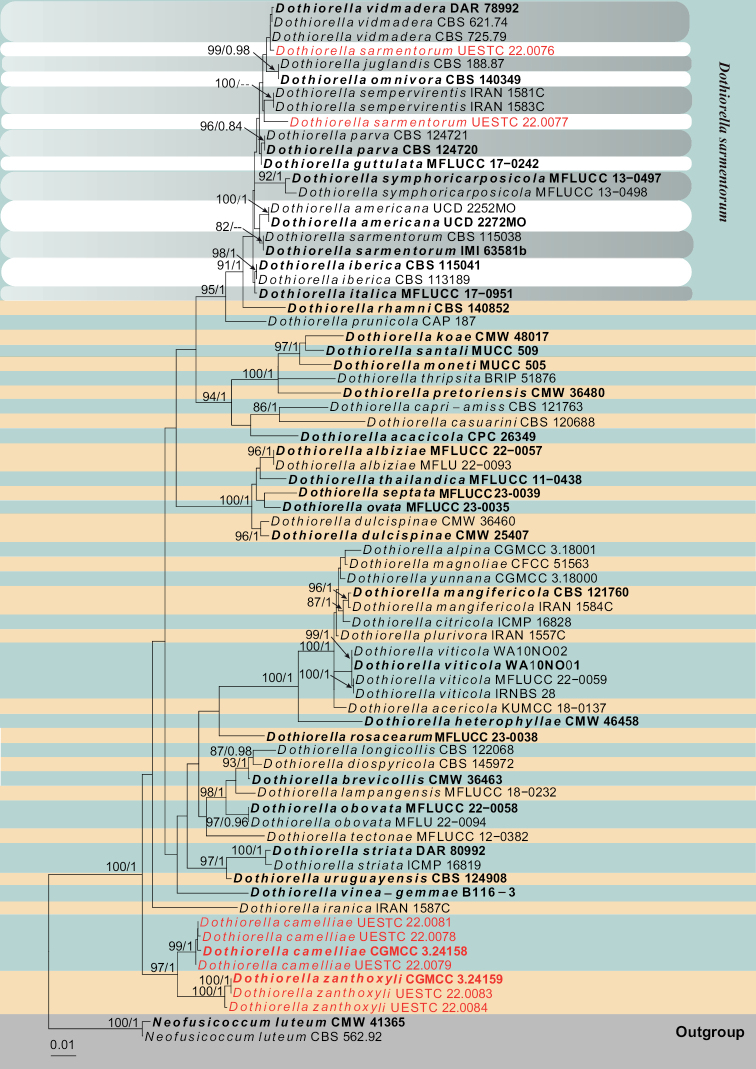
Phylogram generated from RAxML analysis based on combined ITS, *tef1* and *tub2* sequence data of *Dothiorella* isolates. The tree was rooted to *Neofusicoccumluteum* (CBS 562.92 and CMW 41365). The ML (≥ 75%) and BI (≥ 95%) bootstrap supports are given near the nodes, respectively. Isolates from this study are marked in red and ex-type strains are marked in bold.

Three individual phylogenetic trees were constructed for the genera *Botryosphaeria*, *Diplodia* and *Dothiorella*. Twelve isolates belonged to the genus *Botryosphaeria* and ten of them were nested with *Bo.dothidea*, while the remaining two isolates clustered with *Bo.fabicerciana* (Fig. 2). Another twelve isolates were treated in *Diplodia* and seven isolates were clustered with two known species of *Diplodia* (*Di.mutila* and *Di.seriata*, Fig. 3). The other five isolates did not cluster with any previously known *Diplodia* species, thus, two novel species were preliminarily identified based on phylogenetic evidence. Eight isolates were nested within *Dothiorella* and six isolates of them were occupied in the basal position of the *Dothiorella* tree and formed two well‐supported subclades, representing two new species. The other two isolates were nested within the *Do.sarmentorum* isolates (Fig. 4).

### ﻿Taxonomy

#### ﻿ Aplosporella ginkgonis

Taxon classificationFungiBotryosphaerialesBotryosphaeriaceae

C.M. Tian, Z. Du & K.D. Hyde. Mycosphere 8(2): 1249 (2017).

6D8A1E3F-F7BE-55D1-80D5-787E2182406F

 552938

Fig. 5

##### Description.

*Saprobic* on decaying branches of *Zanthoxylumbungeanum*. **Sexual morph**: Not observed. **Asexual morph**: Coelomycetous, ***Conidiomata*** 558–657 × 216–241 μm (*x̄* = 235.5 × 228.5 μm, n = 10), immersed, partially erumpent when mature, multilocular, locules separated by pale brown cells of ***textura angularis*. *Peridium*** 65–106 μm wide, wall 6–10 cell-layers thick, outer layers composed of 3–4 layers of pale brown cells of ***textura globulosa***, intermediate layers composed of dark brown cells of ***textura angularis***, becoming pale brown towards the inner region. ***Ostiole*** 138–171 μm diam., central. ***Conidiophores*** reduced to conidiogenous cells. ***Conidiogenous cells*** 12–13 × 7.5–8 μm (*x̄* = 12.5 × 8 μm, n = 20), holoblastic, hyaline, cylindrical to doliiform, smooth-walled. ***Conidia*** 17–20 × 6.5–7.5 μm (*x̄* = 18.5 × 7 μm, n = 30), L/W ratio = 2.5, ellipsoidal to subcylindrical, with both ends rounded, initially hyaline, becoming dark brown, aseptate.

**Figure 5.**
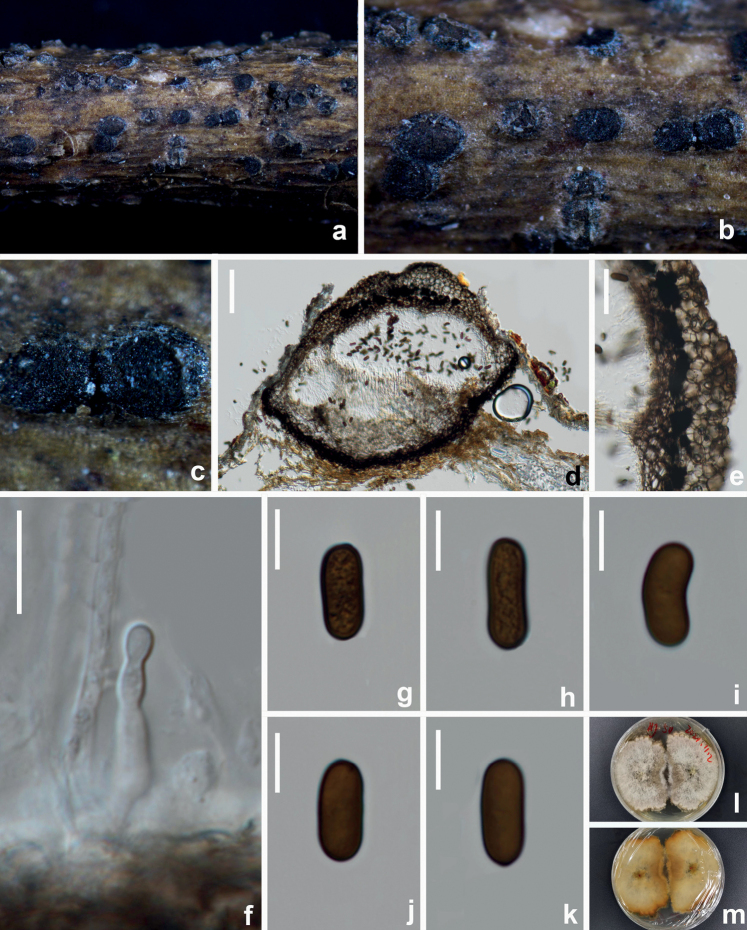
*Aplosporellaginkgonis* (HUEST 22.0092, new host record) **a–c** appearance of conidiomata on natural substrate **d** vertical section of conidioma **e** section of peridium **f** conidiogenous cells and developing conidia **g–k** brown aseptate conidia **l** upper view of the colony on PDA after 14 d **m** reverse view of the colony on PDA after 14 d. Scale bars: 100 μm (**d**); 40 μm (**e**); 10 μm (**f–k**).

##### Culture characteristics.

Colonies on PDA developing dense aerial mycelium with age, becoming white to gray-brown at the surface, and whitish to yellowish brown at the reverse, producing a brown pigment, with sinuate edges.

##### Material examined.

China, Sichuan Province, Yaan City, Hanyuan County, 29°16'51"N, 102°37'48"E, elevation 1,689 m, on dead branches of *Zanthoxylumbungeanum*, 30^th^ October 2021, W.L. Li, HJ 511 (HUEST 22.0092), living culture UESTCC 22.0091.

##### Notes.

*Aplosporellaginkgonis* was introduced by Du et al. (2017) and isolated from diseased branches of *Ginkgobiloba* and *Morusalba* from Gansu Province in China. One isolate (UESTCC 22.0091) obtained in this study from *Zanthoxylumbungeanum* is morphologically similar to the original description of *Aplosporellaginkgonis*, and the sequences data are identical to the previous data (99%–100%). We, thus, identify the new collection as *Aplosporellaginkgonis* and this is the first report from *Zanthoxylumbungeanum*.

#### ﻿ Aplosporella prunicola

Taxon classificationFungiBotryosphaerialesBotryosphaeriaceae

Damm & Crous Fungal Diversity 27: 39 (2007).

F381966D-E265-555A-9986-B2F5712013BD

 504373

Fig. 6

##### Description.

*Saprobic* on decaying branches of *Zanthoxylumbungeanum*. **Sexual morph**: Not observed. **Asexual morph**: Coelomycetous, ***Conidiomata*** 355–408 × 568.5–599 μm (*x̄* = 381.5 × 584 μm, n = 10), immersed, partially erumpent when mature, multilocular, locules divided by pale brown cells of ***textura angularis*. *Peridium*** 107–122 μm wide, composed of 3–5 layers of pale brown cells of ***textura globulosa*. *Ostiole*** 70–88 μm diam., central. ***Conidiophores*** reduced to conidiogenous cells. ***Conidiogenous cells*** 6.5–10 × 2–3 μm (*x̄* = 8 × 2.5 μm, n = 20), holoblastic, hyaline, cylindrical, smooth-walled. ***Conidia*** 20–23.5 × 12–13.5 μm (*x̄* = 21.5 × 13 μm, n = 30), L/W ratio = 1.6, ellipsoidal to subcylindrical, with both ends broadly rounded, initially hyaline, becoming dark brown, aseptate, smooth.

**Figure 6.**
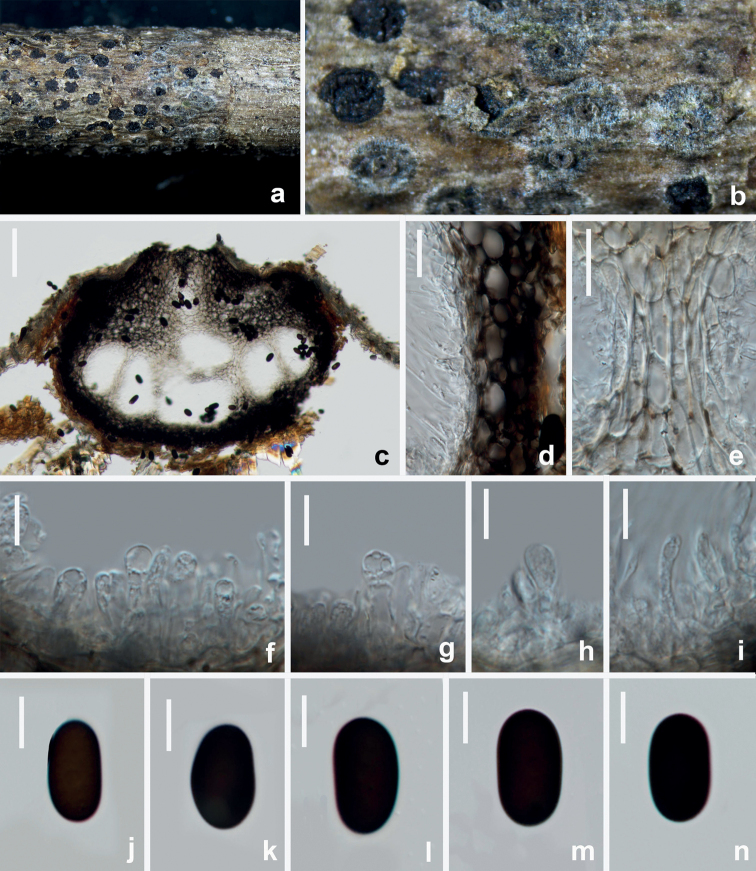
*Aplosporellaprunicola* (HUEST 22.0091, new host record) **a, b** appearance of conidiomata on natural substrate **c** vertical section of multiloculate conidioma **d, e** section of peridium **f–i** conidiogenous cells and developing conidia **j–n** brown aseptate conidia. Scale bars: 100 μm (**c**); 20 μm (**d, e**); 10 μm (**f–n**).

##### Culture characteristics.

Colonies on PDA after 7 d, becoming pale olivaceous-gray to olivaceous-black at the surface, and olivaceous black at the reverse, with irregular edges.

##### Material examined.

China, Sichuan Province, Yaan City, Hanyuan County, 29°16'51"N, 102°37'48"E, elevation 1,689 m, on dead branches of *Zanthoxylumbungeanum*, 30^th^ October 2021, W.L Li, HJ 509 (HUEST 22.0091), living culture UESTCC 22.0090.

##### Notes.

Our isolate UESTCC 22.0090 morphologically lines up with the description of *Aplosporellaprunicola* provided by Damm et al. (2007) in having immersed to erumpent, multilocular conidiomata and brown, smooth‐walled, ovoid to oblong conidia. The strain UESTCC 22.0090 is phylogenetically and morphologically similar to *A.yalgorensis* and *A.prunicola*, however, *A.yalgorensis* can be distinguished from other *Aplosporella* species by its pitted conidial walls. Thus, the strain UESTCC 22.0090 was identified as *A.prunicola* based on current evidence. This is the first time *A.prunicola* is reported from *Zanthoxylumbungeanum* in China.

#### ﻿ Diplodia acerigena

Taxon classificationFungiBotryosphaerialesBotryosphaeriaceae

L.W. Li & Jian K. Liu sp. nov.

A5B7C2CA-507C-5B7A-A8C9-391CFD522891

 847163

Figs 7 , 8

##### Etymology.

The epithet ‘‘acerigena’’ refers to the host genus *Acer*, on which the holotype was collected.

##### Holotype.

HKAS 125891.

##### Description.

*Saprobic* on decaying branches of *Acertruncatum*. **Sexual morph: *Ascomata*** 304.5–321 × 217–260 (*x̄* = 313 × 238.5 μm, n = 20), more or less subglobose, solitary or gregarious, semi-immersed, medium brown to dark brown, unilocular, papillate, ostiolate. ***Ostiole*** 101–115 μm diam., conical or circular, central, papillate, periphysate. ***Peridium*** 23–29 μm wide, composed of 3–5 layers of dark brown cells of ***textura angularis*. *Pseudoparaphyses*** 3.5–5 μm wide, hyaline, branched, septate. ***Asci*** 98–120 × 24–32.5 μm (*x̄* = 109 × 28 μm, n = 30), (4–)8-spored, clavate, stipitate, irregularly bitunicate, apex rounded with an ocular chamber. ***Ascospores*** 24.5–31.5 × 13.5–16 μm (*x̄* = 28 × 14.5 μm, n = 30), L/W ratio = 2, biseriate, broadly fusiform to oval, widest in the middle, both ends obtuse, hyaline, moderately thick-walled, smooth, becoming brown and 2-septate when aged. **Asexual morph**: Coelomycetous, pycnidia produced on mycelium in PDA. ***Conidiomata*** stromatic, mostly solitary, gray to black, globose to subglobose. ***Paraphyses*** 2–3.5 μm wide, hyaline, subcylindrical, branched, septate. ***Conidiophores*** absent. ***Conidiogenous cells*** 9–12 × 3.5–5 μm (*x̄* = 10.5 × 4.5 μm, n = 20), holoblastic, hyaline, cylindrical. ***Conidia*** 21–24 × 10–11 μm (*x̄* = 22.5 × 10.5 μm, n = 30), L/W ratio = 2, aseptate, thick-walled, wall externally smooth, roughened on the inner surface, initially hyaline becoming dark brown, obovoid to ellipsoid, both ends broadly rounded. ***Spermatogenous cells*** 7–9.5 × 2.5–3.5 μm (*x̄* = 8 × 3 μm, n = 20), discrete or integrated, hyaline, smooth, cylindrical, holoblastic or proliferating via. determinate phialides with periclinal thickening. ***Spermatia*** 7–11.5 × 3–4 μm (*x̄* = 9 × 3.5 μm, n = 30), hyaline, smooth, aseptate, rod-shaped with rounded ends.

**Figure 7.**
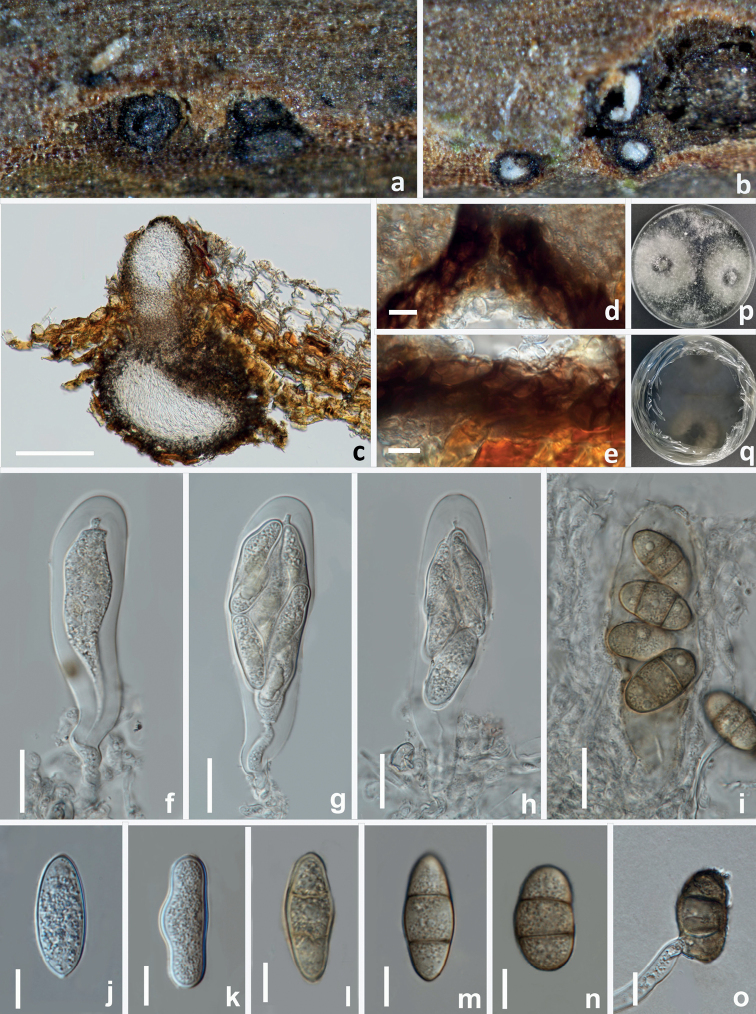
The sexual morph of *Diplodiaacerigena* (HKAS 125891, holotype) **a, b** appearance of ascomata on natural substrate **c** vertical section of ascoma **d** ostiole **e** section of peridium **f–h** asci with hyaline ascospores **i** asci with brown 2-sepatate ascospores **j, k** hyaline immature aseptate ascospores **l–n** mature brown 2-septate ascospores **o** germinated ascospore **p** upper view of the colony on PDA after 14 d **q** reverse view of the colony on PDA after 14 d. Scale bars: 100 μm (**c**); 10 μm (**d, e, j–o**); 20 μm (**f–i**).

##### Culture characteristics.

Ascospores germinating on PDA within 12 h. Colonies growing on PDA, reaching a diam. of 4 cm after five days at 25 °C, effuse, velvety, with entire to slightly undulate edge. Surface initially white and later turning dark olivaceous from the surrounding of the colony and dark gray in reverse.

**Figure 8.**
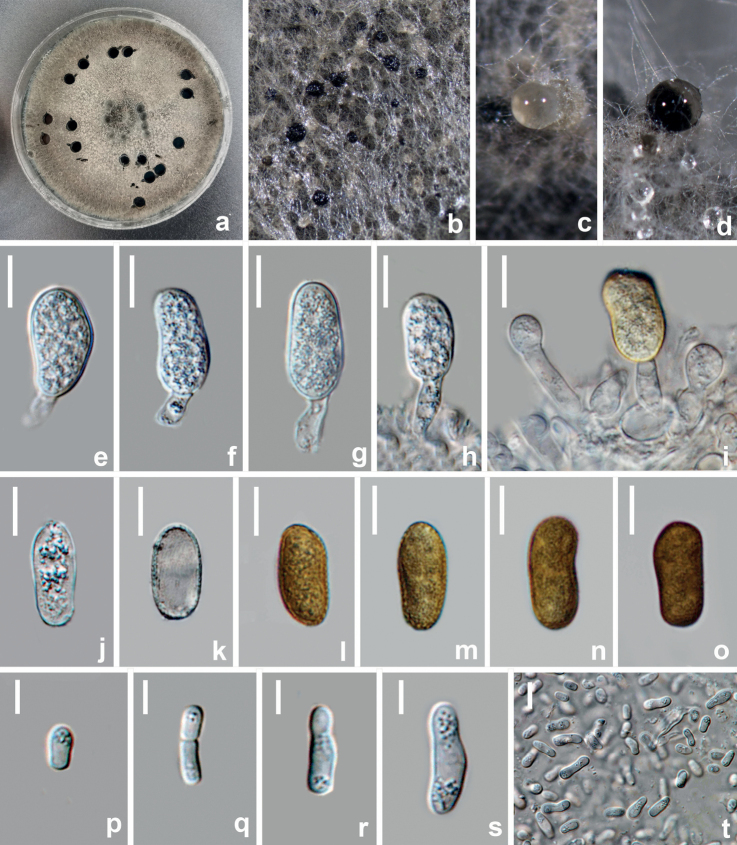
The asexual morph of *Diplodiaacerigena* (HKAS 125891, holotype) **a–d** appearance of conidiomata on PDA**e–i** conidiogenous cells and developing conidia **j, k** hyaline immature conidia **l–o** mature brown aseptate conidia **p–t** Spermatogenous cells and Spermatia. Scale bars: 10 μm (**e–o, t**); 5 μm (**p–s**).

##### Material examined.

China, Sichuan Province, Chengdu City, Pidu District, 30°19'57"N, 103°59'47"E, elevation 442 m, on dead branches of *Acertruncatum* (Anacardiaceae), 19^th^ March 2021, W.L Li, YBF 96 (HKAS 125891, holotype), ex-type living culture UESTCC 22.0073 = CGMCC 3.24157; *ibid*., YBF103 (HUEST 22.0075, paratype), living culture UESTCC 22.0074. Additional sequences: LSU: OQ164827 (CGMCC 3.24157), OQ164828 (UESTCC 22.0074).

##### Notes.

Three isolates of *Diplodiaacerigena* clustered closer to *Di.pseudoseriata* (CBS 124906) with high bootstrap support (ML/BI 100%/1). The asexual morph of *Diplodiapseudoseriata* was introduced by Pérez et al. (2010), collected and isolated from the *Blepharocalyxsalicifolius* in Uruguay and its sexual morph has not been reported. The asexual morph of *Diplodiaacerigena* differs from *Di.pseudoseriata* in having conidia which become 1-septate when aged. *Diplodiaacerigena* shares similar sexual morph characters as of other *Diplodia* species by having immersed to semi-immersed pseudothecia, clavate asci, broadly fusiform to ovoid and hyaline ascospores. However, conidia of *Diplodiaacerigena* become brown and septate when aged, which is rarely observed in any other sexual morph species of this genus.

#### ﻿ Diplodia mutila

Taxon classificationFungiBotryosphaerialesBotryosphaeriaceae

(Fr.) Mont., Ann. Sci. nat., sér. 2, 1: 302. 1834.

C48DAD9D-35D2-5A27-B79C-A6B6033197E8

 201741

Fig. 9

Sphaeria mutila Fr., Syst. Mycol. (Lundae) 2: 424. 1823. Basionym.
≡ Physalosporamutila (Fr.) N.E. Stevens, Mycologia 28: 333. 1936.
= Botryosphaeriastevensii Shoemaker, Canad. J. Bot. 42: 1299. 1964.

##### Description.

*Saprobic* on decaying branches of *Camelliaoleifera*. **Sexual morph**: Not observed. **Asexual morph**: Coelomycetous, ***Conidiomata*** 330–394 × 215–230 μm (*x̄* = 362 × 223 μm, n = 10), immersed, erumpent, gregarious, dark brown to black, subglobose, unilocular. ***Ostiole*** 48.5–67 μm diam., central. ***Peridium*** 29–38 μm wide, thick-walled, outer and inner layers composed of 1–2 layers dark brown ***textura angularis***, intermediate layers composed of 3–5 layers of hyaline cells of ***textura angularis*. *Conidiophores*** reduced to conidiogenous cells. ***Conidiogenous cells*** 8.5–12 × 3–5 μm (*x̄* = 10 × 4 μm, n = 20), cylindrical, thin-walled, hyaline, holoblastic, indeterminate, proliferating at the same level to produce periclinal thickenings, or proliferating percurrently giving rise to 2–3 indistinct annellations. ***Conidia*** 19–21 × 9.5–11 μm (*x̄* = 20 × 10.5 μm, n = 30), L/W ratio = 2, oblong, with broadly rounded apex and truncate base, thick-walled, wall externally smooth, roughened on the inner surface, hyaline, aseptate, becoming dark brown when aged.

**Figure 9.**
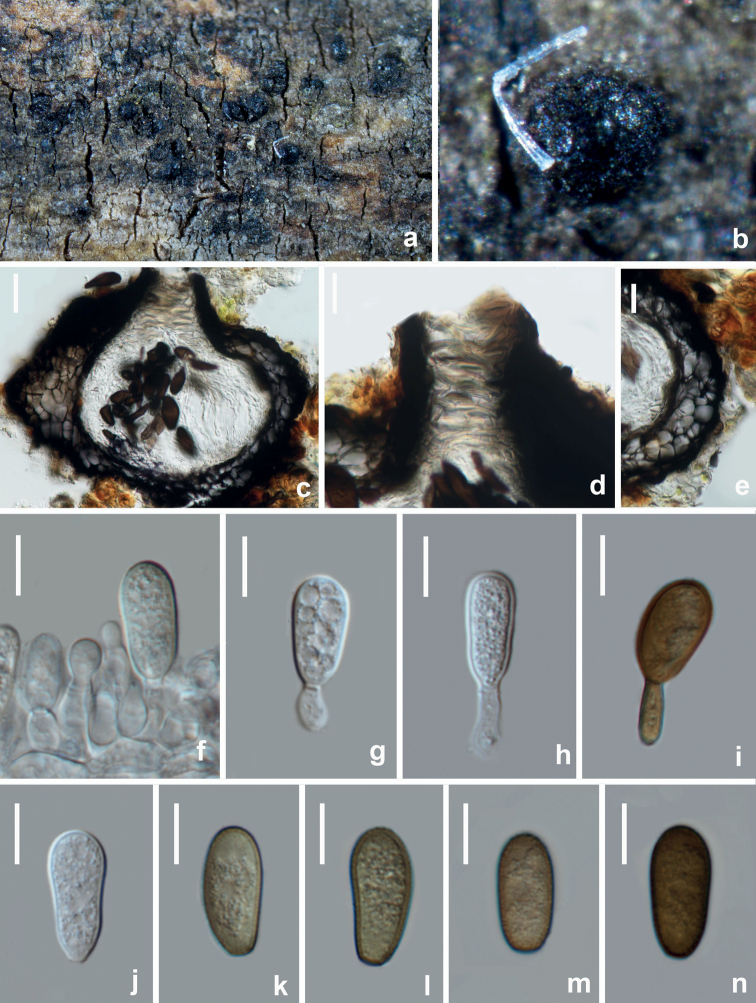
*Diplodiamutila* (HUEST 22.0069, new host record) **a, b** appearance of conidiomata on natural substrate **c** vertical section of conidioma **d** ostiole **e** section of peridium **f–i** conidiogenous cells and developing conidia **j** hyaline immature conidium **k–n** mature brown conidia. Scale bars: 40 μm (**c**); 20 μm (**d, e**); 10 μm (**f–n**).

##### Culture characteristics.

Colonies on PDA initially olivaceous buff in the center of the colony and white at the edge, becoming olivaceous within 7 d on the surface, with smooth edge.

##### Materials examined.

China, Sichuan Province, Jiangyou City, Shuanghe County, 31°54'10"N, 104°55'57"E, elevation 657 m, on dead branches of *Camelliaoleifera*, 11^th^ July 2021, W.L Li, 286 (HUEST 22.0069), living culture UESTCC 22.0068; *ibid*., 289 (HUEST 22.0068), living culture UESTCC 22.0067; *ibid*., Guangyuan city, Qingchuan County, 32°40'38"N, 105°28'57"E, elevation 634 m, on dead branches of *Oleaeuropaea*, 20^th^ April 2021, W.L Li, 188 (HUEST 22.0065), living culture UESTCC 22.0064; *ibid*., 257 (HUEST 22.0070), living culture UESTCC 22.0069; *ibid*., on dead branches of *Verniciafordii*, 20^th^ April 2021, W.L Li, 238 (HUEST 22.0066), living culture UESTCC 22.0065; *ibid*., Chengdu City, Pidu District, 30°49'27"N, 103°47'42"E, elevation 442 m, on dead branches of *Pistaciachinensis*, 5^th^ March 2021, W.L Li, A61 (HUEST 22.0064), living culture UESTCC 22.0063. Additional sequences: LSU: OQ164832 (UESTCC 22.0063), OQ164830 (UESTCC 22.0064), OQ164831 (UESTCC 22.0065).

##### Notes.

The phylogenetic tree show that six strains isolated from *Camelliaoleifera*, *Oleaeuropaea* and *Verniciafordii* nested with *Diplodiamutila* (CBS 112553) with a moderate bootstrap support (ML/BI 86%/1). *Diplodiamutila*, the type of the genus, is a well-known and most commonly reported species. It has been recorded mainly from woody substrates, and it is known from more than 50 hosts (Batista et al. 2021). Morphologically, one of the isolates obtained in this study UESTCC 22.0068 shares similar conidia shape and size with *Di.mutila*, but hardly observed the mature conidia with septa. We identify these taxa as *Di.mutila* based on morphology and phylogeny evidences. This is the first report of *Di.mutila*, isolated from *Camelliaoleifera*, *Oleaeuropaea* and *Verniciafordii*.

#### ﻿ Diplodia pistaciicola

Taxon classificationFungiBotryosphaerialesBotryosphaeriaceae

L.W. Li & Jian K. Liu sp. nov.

0A8A7606-E2F6-5AC0-AFE4-A94CE7163BAD

 847166

Fig. 10

##### Etymology.

The epithet ‘‘*pistaciicola*’’ refers to the host genus *Pistacia*, on which the holotype was collected.

##### Holotype.

HKAS 125890.

##### Description.

*Saprobic* on decaying branches of *Pistaciachinensis*. **Sexual morph**: Not observed. **Asexual morph**: Coelomycetous, ***Conidiomata*** 353–441 × 274.5–316 μm (*x̄* = 397 × 295 μm, n = 10), immersed, forming split-like opening on the host, solitary or gregarious, globose to subglobose, dark brown to black, unilocular, papillate, ostiolate. ***Ostiole*** 38–49.5 μm diam., conical or circular, centrically located. ***Peridium*** 42–60 μm wide, composed of thick walled, dark brown to hyaline cells of ***textura angularis*. *Conidiophores*** reduced to conidiogenous cells. ***Conidiogenous cells*** 10–14 × 3–4 μm (*x̄* = 12 × 3.5 μm, n = 20), holoblastic, discrete, cylindrical, hyaline, smooth, indeterminate, arising from the inner cavity of the conidiomata. ***Conidia*** 24.5–27 × 11–13 μm (*x̄* = 25.5 × 12 μm, n = 30), L/W ratio = 2.2, ellipsoid to obovoid, aseptate, hyaline, thick-walled, guttulate.

**Figure 10.**
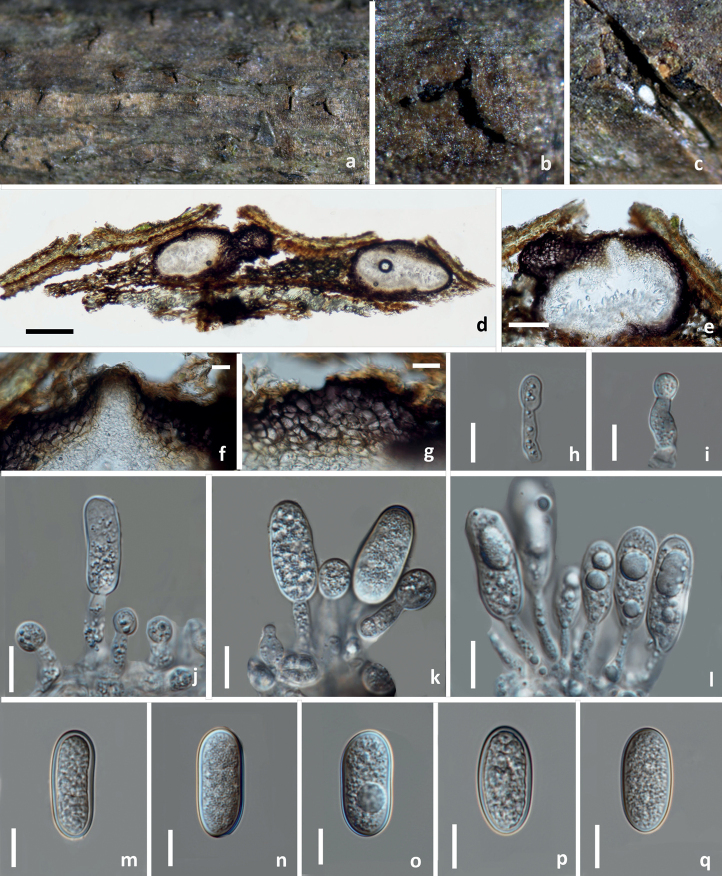
*Diplodiapistaciicola* (HKAS 125890, holotype) **a–c** appearance of conidiomata on natural substrate **d, e** vertical section of conidiomata/conidioma **f** ostiole **g** section of peridium **h–l** conidiogenous cells and developing conidia **m–q** hyaline aseptate conidia. Scale bars: 200 μm (**d**); 50 μm (**e**); 20 μm (**f, g**); 10 μm (**h–q**).

##### Culture characteristics.

Conidia germinate on PDA within 12 h. Colonies growing on PDA, reaching a diameter of 4 cm after five days at 25 °C, effuse, velvety, with entire to slightly undulate edge. The early stage of the colony is white, later turning dark olivaceous and dark gray in reverse.

##### Material examined.

China, Sichuan Province, Chengdu City, Pidu District, 30°49'27"N, 103°47'42"E, elevation 442 m, on dead branches of *Pistaciachinensis* (Anacardiaceae), 5^th^ March 2021, W.L Li, 049 (HKAS 125890, holotype), ex-type living culture UESTCC 22.0070 = CGMCC 3.24156; *ibid*., 049B (HUEST 22.0072 isotype), ex-isotype living culture UESTCC 22.0071. Additional sequence: LSU: OQ164833 (CGMCC 3.24156).

##### Notes.

Phylogenetic analyses showed that two strains of *Diplodiapistaciicola* isolated from *Pistaciachinensis* are distinct but closely related to *Di.agrifolia* (CBS 124.30). The comparison of ITS, *tef1* and *tub2* of these two species indicate 5 bp (502), 3bp (224), 9 bp (425) differances, respectively. Morphologically, *Di.agrifolia* differs from *Di.pistaciicola* in producing two to three times larger ascomata than that of *Di.pistaciicola* (721–836 vs. 274.5–316 μm) and possessing smaller conidia (27–36.5 × 14.5–17.8 μm vs. 24.5–27 × 11–13 μm). In addition, conidia of *Di.pistaciicola* are hyaline, aseptate, rarely becoming pale brown and uniseptate with age, whereas conidia of *Di.agrifolia* are mostly dark brown and uniseptate before discharge from pycnidia.

#### ﻿ Diplodia seriata

Taxon classificationFungiBotryosphaerialesBotryosphaeriaceae

De Not., Micr. Ital. Dec. 4: 6. (1942).

68B64311-7876-50D0-A26B-39D963E2FB8D

 180468

Fig. 11

##### Description.

*Saprobic* on decaying branches of *Camelliaoleifera*. **Sexual morph: *Ascomata*** 301–343 × 293–340 (*x̄* = 322 × 316 μm, n = 10), more or less subglobose, solitary or gregarious, semi-immersed, medium brown to dark brown, unilocular, papillate, ostiolate. ***Ostiole*** 72–78 μm diam., conical or circular, central, papillate, periphysate. ***Peridium*** 33–44 μm wide, composed of dark brown, 4–6 layers of ***textura angularis*. *Pseudoparaphyses*** 2–2.5 μm wide, hyaline, branched, septate. ***Asci*** 112–141 × 27.5–30 μm (*x̄* = 126 × 28.5 μm, n = 30), clavate, stipitate, bitunicate, containing eight, biseriate ascospores. ***Ascospores*** 31.5–32.5 × 12–13.5 μm (*x̄* = 32 × 13 μm, n = 30), L/W ratio = 2.5, broadly fusiform to oval, widest in the middle, both ends obtuse, hyaline, moderately thick-walled, smooth, becoming brown when aged. **Asexual morph**: Not observed.

**Figure 11.**
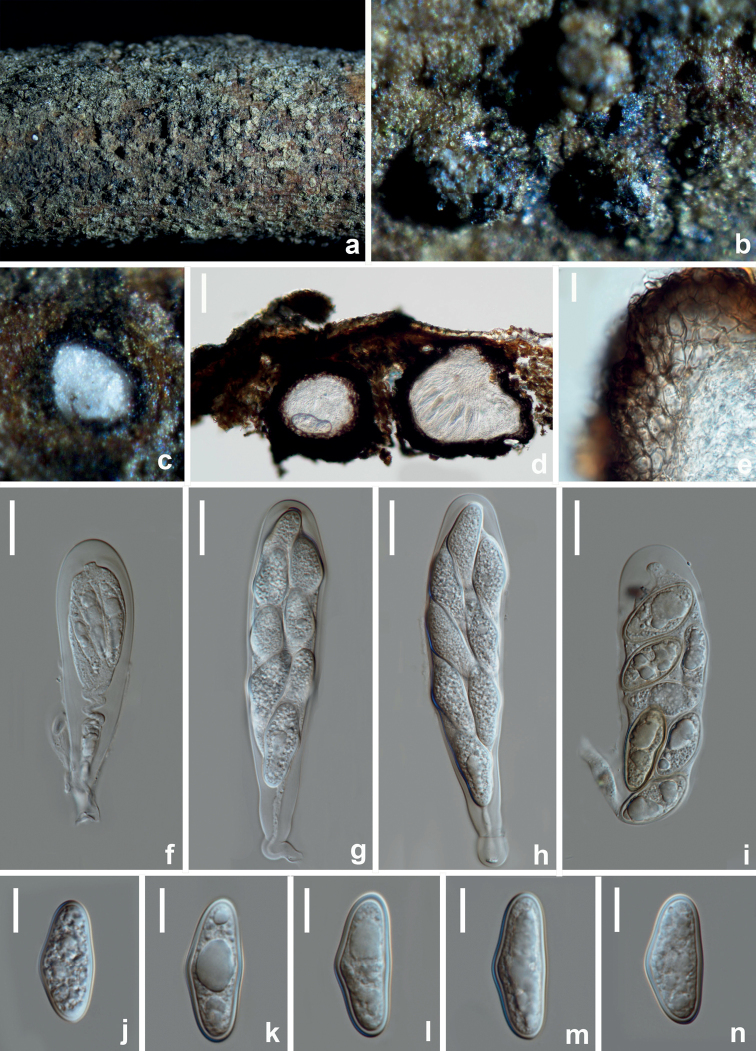
*Diplodiaseriata* (HUEST 22.0073, new host record) **a–c** appearance of ascomata on natural substrate **d** vertical section of ascomata **e** section of peridium **f–i** asci **j–n** ascospores. Scale bars: 100 μm (**d**); 10 μm (**e, j–n**); 20 μm (**f–i**).

##### Culture characteristics.

Ascospores germinate on PDA within 12 h. Colonies growing on PDA, reaching a diameter of 4 cm after five days at 25 °C, effuse, velvety, with entire to slightly undulate edge.

##### Material examined.

China, Sichuan Province, Jiangyou City, shuanghe County, 31°54'10"N, 104°55'57"E, elevation 656 m, on dead branches of *Camelliaoleifera*, 10^th^ June 2021, W.L Li, 288 (HUEST 22.0073), living culture UESTCC 22.0072.

##### Notes.

The morphology of the taxa isolated from decaying woody oil plants is similar to *Diplodiaseriata*. In the multi-gene phylogenetic analysis, our new collection clustered with the ex-type strain of *Di.seriata* (CBS 112555) with strong bootstrap support. *Diplodiaseriata* has been isolated from a wide range of hosts (121 species) and has a worldwide distribution (reported in 46 countries) (Batista et al. 2021). This is the first report of *Di.seriata* isolated from *Camelliaoleifera*.

#### ﻿ Dothiorella camelliae

Taxon classificationFungiBotryosphaerialesBotryosphaeriaceae

L.W. Li & Jian K. Liu sp. nov.

637B8257-D8BF-5044-9FBB-709E0C78DB1E

 847167

Fig. 12

##### Etymology.

The epithet ‘‘*camelliae*’’ refers to the host genus *Camellia*, on which the holotype was collected.

##### Holotype.

HKAS 125892.

##### Description.

*Saprobic* on decaying branches of *Camelliaoleifera*. **Sexual morph: *Ascomata*** 199–222 × 237–269 μm (*x̄* = 210.5 × 253 μm, n = 10), submerged in the substrate, partly erumpent at maturity, solitary or gregarious, dark brown to black, subglobose, multilocular or unilocular. ***Ostiole*** 17–37 μm diam., central. ***Peridium*** 35–43 μm wide, thick-walled, outer layers composed of 1–2 layers dark brown cells of ***textura angularis***, becoming hyaline towards the inner region. ***Pseudoparaphyses*** 3–4 μm wide, hyaline, frequently aseptate. ***Asci*** 80–96 × 22–25 μm (*x̄* = 88 × 23.5 μm, n = 30), stipitate, clavate, thick-walled, bitunicate, (6–)8-spored, irregularly biseriate. ***Ascospores*** 21–25 × 9.5–12 μm (*x̄* = 23 × 10.5 μm, n = 30), L/W ratio = 2, oblong, ovate to sub-clavate, (0–)1-septate, slightly constricted at the septum, hyaline to dark brown, moderately thick-walled, straight or inequilateral, basal cell tapering towards the acute end. **Asexual morph**: Not observed.

**Figure 12.**
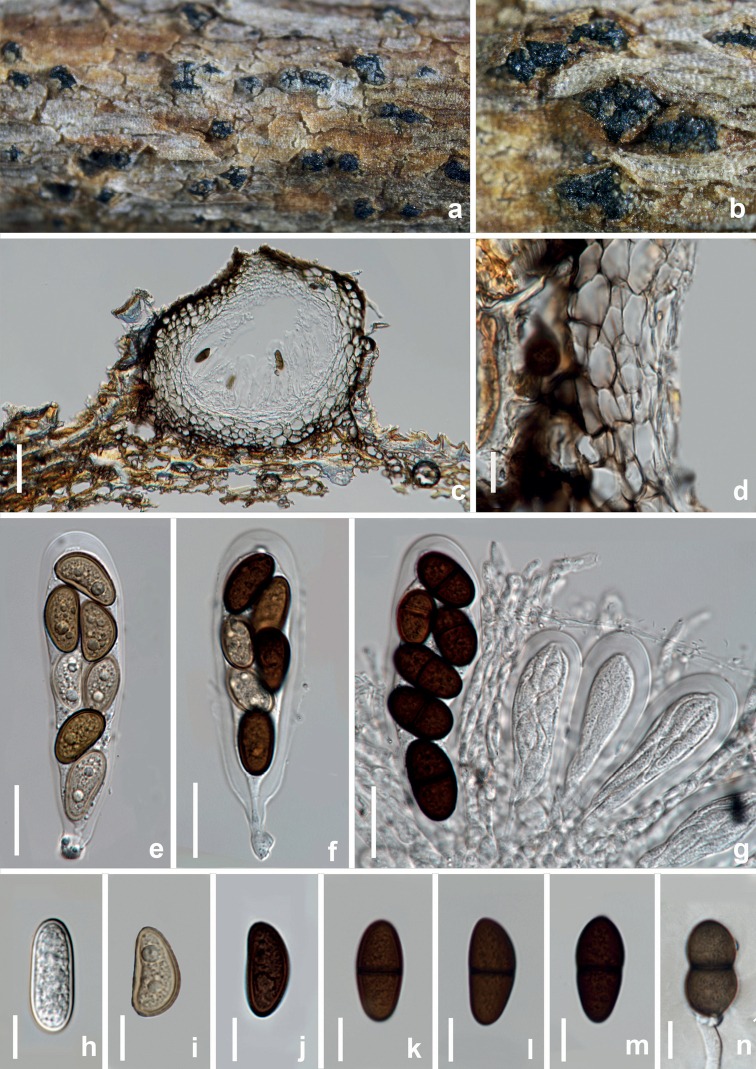
*Dothiorellacamelliae* (HKAS 125892, holotype) **a, b** appearance of ascomata on natural substrate **c** vertical section of ascoma **d** section of peridium **e–g** asci **h–m** ascospores **n** germinated ascospore. Scale bars: 50 μm (**c**); 10 μm (**d, h–n**); 20 μm (**e–g**).

##### Culture characteristics.

Ascospores germinate on PDA within 12 h. Colonies growing on PDA, reaching a diameter of 4 cm after five days at 25 °C, effuse, velvety, with entire to slightly undulate edge. Surface initially white and later turning dark olivaceous from the surrounding of the colony and dark gray in reverse.

##### Materials examined.

China, Sichuan Province, Leshan City, Wutongqiao District, 29°22'28"N, 103°45'49"E, elevation 383 m, on dead branches of *Camelliaoleifera* (Theaceae), 23^th^ July 2021, Z.P Liu, 351 (HKAS 125892, holotype), ex-type living culture UESTCC 22.0081 = CGMCC 3.24158; *ibid*., 347 (HUEST 22.0081), living culture UESTCC 22.0080; *ibid*., Shizhong District, 29°42'13"N, 103°52'25"E, elevation 356 m, on dead branches of *Paeoniasuffruticosa*, 23^th^ July 2021, W.L Li, A240 (HUEST 22.0080), living culture UESTCC 22.0079; *ibid*., A234 (HUEST 22.0079), living culture UESTCC 22.0078. Additional sequences: LSU: OQ164834 (CGMCC 3.24158), OQ164835 (UESTCC 22.0079), OQ164836 (UESTCC 22.0078).

##### Notes.

Four strains isolated from *Verniciafordii* and *Camelliaoleifera* occupy a basal position in the *Dothiorella* phylogenetic tree by forming a well‐supported subclade sister to *Do.zanthoxyli* (ML/BI 97%/1, Fig. 4). The BLASTn searches of the ITS sequence of *Dothiorellazanthoxyli* resulted in 97% matches with *Neofusicoccumvitifusiforme* BRIP64010, the *tef1* showed 91.23% matches with *Do.symphoricarposicola* BL158, and the *tub2* BLASTn results indicated 96.53% similarity with *Do.uruguayensis* CBS 124908 and *Do.viticola* B116-3. *Dothiorellacamelliae* can be distinguished from *Do.zanthoxyli* in the size of ascomata, ascus and L/W ratio of ascospores (Table 3). *Dothiorellacamelliae* resembles the sexual morph of *Do.sarmentorum* in producing immersed to sub-immersed ascomata, clavate asci and ovate to sub-clavate, hyaline to brown conidia with (0–)1-septate. However, *Do.sarmentorum* morphologically can be distinguished from *Do.camelliae* in having larger ascomata (350–400 μm vs. 237–269 μm), thicker peridium (50–75 μm vs. 35–43 μm), and longer asci (140–210 μm vs. 80–96 μm) (Table 3). Phylogenetically, these two species reside in two distinct clades.

**Table 3.** A morphological comparison of the sexual morph of three *Dothiorella* species.

Taxa	Ascomata (μm)	Asci (μm)	Peridium (μm)	Ascospores		
				Size(μm)	Color	L/W ratio
*Dothiorellacamelliae*	199–222 × 237–269	80–96 × 22–25	35–43	21–25 × 9.5–12	Hyaline to dark brown	2
*Dothiorellasarmentorum*	350–400	140–210 × 17–24	50–75	24.5–25.5 × 11.5–12.5	Dark brown	2.4
*Dothiorellazanthoxyli*	258–280 × 170–174	63.5–77 × 20–24.5	35–40	22.5–25 × 9.5–11	Hyaline to dark brown	2.6

#### ﻿ Dothiorella sarmentorum

Taxon classificationFungiBotryosphaerialesBotryosphaeriaceae

(Fr.) A.J.L. Phillips, J. Luque & A. Alves, Mycologia 97: 522. (2005).

75A90425-ABC3-57FC-BD0C-63A8B6A81076

 501403

Fig. 13

Sphaeria sarmentorum Fr., K. svenska Vetensk-Acad. Handl. 39: 107. 1818. Basionym.
≡ Diplodiasarmentorum (Fr.) Fr., Summ. veg. Scand. (Stockholm) 2: 417. 1849.
= Diplodiapruni Fuckel, Jahrb. Nassauischen Vereins Naturk., 23–24: 169. 1870 [1869].
= Botryosphaeriasarmentorum A.J.L. Phillips, J. Luque & A. Alves, Mycologia 97: 522. 2005.

##### Description.

*Saprobic* on decaying branches of *Pistaciachinensis*. **Sexual morph**: Not observed. **Asexual morph: *Conidiomata*** 278–338 × 240–280 μm (*x̄* = 308 × 260 μm, n = 10), immersed, erumpent, forming split-like opening on the host, gregarious, globose to subglobose, dark brown to black, unilocular or multilocular, papillate, ostiolate. ***Ostiole*** 52–57 μm diam., conical or circular, centrically located. ***Peridium*** 28.5–44 μm, comprising 5–8 layers of thick‐walled, dark brown to hyaline cells arranged in a ***textura angularis*. *Conidiophores*** reduced to conidiogenous cells. ***Conidiogenous cells*** 2.5–3.5 × 6–9 μm (*x̄* = 3 × 7.5 μm, n = 20), holoblastic, discrete, cylindrical, hyaline, smooth, indeterminate, proliferating at the same level giving rise to periclinal thickenings, or rarely proliferating percurrently to form one or two close, indistinct annellations. ***Conidia*** 21.5–24 × 9–10 μm (*x̄* = 22.5 × 9.5 μm, n = 30), L/W ratio = 2.4, ellipsoid to obovoid, with rounded ends, initially hyaline and aseptate becoming pigmented brown and 1-septate often while still attached to conidiogenous cell, brown walled, slightly constricted at the septum.

**Figure 13.**
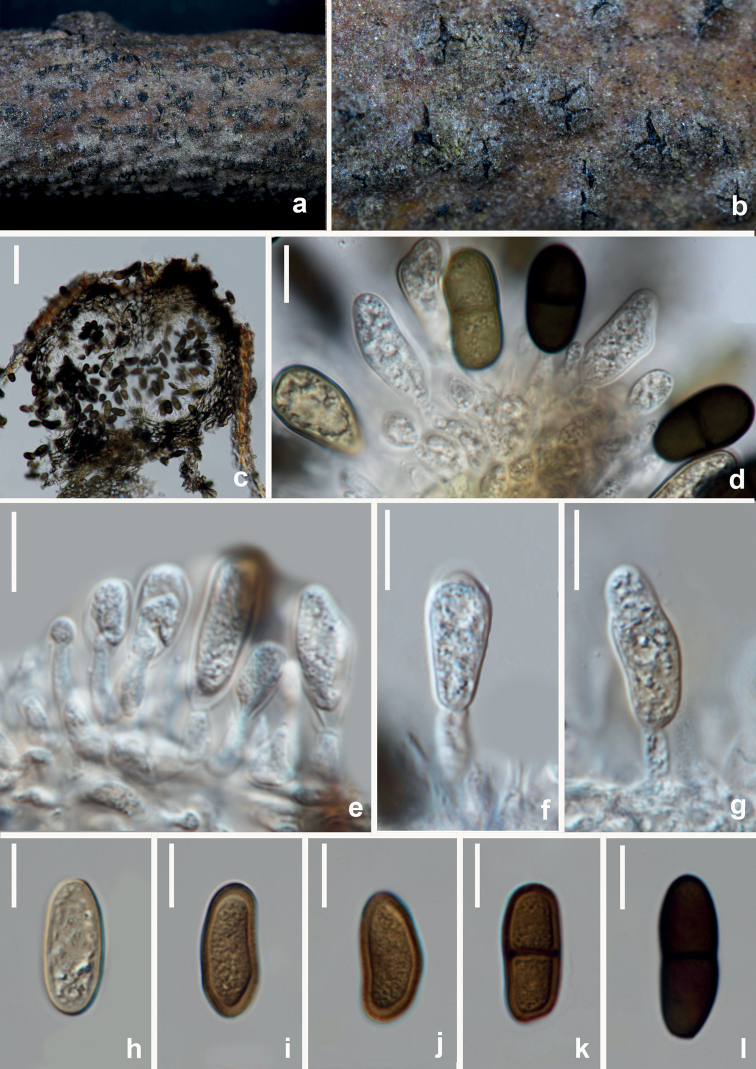
*Dothiorellasarmentorum* (HUEST 22.0077, new host record) **a, b** appearance of conidiomata on natural substrate **c** vertical section of conidioma **d–g** conidiogenous cells and developing conidia **h–l** brown conidia. Scale bars: 50 μm (**c**); 10 μm (**d–l**).

##### Culture characteristics.

Conidia germinate on PDA within 12 h. Colonies growing on PDA, reaching a diameter of 4 cm after three days at 25 °C, effuse, velvety, with entire to slightly undulate edge. Surface initially white and later turning dark olivaceous from the surrounding of the colony and dark gray in reverse.

##### Materials examined.

China, Sichuan Province, Chengdu City, Pidu District, 30°19'57"N, 103°59'47"E, elevation 442 m, on dead branches of *Pistaciachinensis*, 19^th^ March 2021, W.L Li, 072 (HUEST 22.0077), living culture UESTCC 22.0076; *ibid*., Guangyuan City, Qingchuan County, 32°40'38"N, 105°28'57"E, elevation 638 m, 20^th^ April 2021, W.L Li, A189 (HUEST 22.0078), living culture UESTCC 22.0077. Additional sequences: LSU: OQ164837 (UESTCC 22.0076), OQ164838 (UESTCC 22.0077).

##### Notes.

*Dothiorellasarmentorum* was introduced by Phillips et al (2005) with both asexual and sexual morphs. Recently, nine *Dothiorella* species (*Do.californica*, *Do.iberica*, *Do.italica*, *Do.guttulata*, *Do.omnivora*, *Do.parva*, *Do.sempervirentis*, *Do.symphoricarpicola*, *Do.vidmadera*) were synonymized under *Do.Sarmentorum* by Zhang et al. (2021) based on phylogenetic analyses. Two isolates obtained in the present study clustered with the group of *Do.sarmentorum* taxa in the phylogenetic analyses (Fig. 4).

#### ﻿ Dothiorella zanthoxyli

Taxon classificationFungiBotryosphaerialesBotryosphaeriaceae

L.W. Li & Jian K. Liu sp. nov.

D5DE5045-63E3-5163-B674-A38A8760953F

 847168

Fig. 14

##### Etymology.

The epithet ‘‘*zanthoxyli*’’ refers to the host genus *Zanthoxylum*, on which the holotype was collected.

##### Holotype.

HKAS 125893.

##### Description.

*Saprobic* on decaying branches of *Zanthoxylumbungeanum*. **Sexual morph: *Ascomata*** 258–280 × 170–174 μm (*x̄* = 269 × 172 μm, n = 10), submerged in the substrate, partly erumpent at maturity, solitary or gregarious, dark brown to black, subglobose, unilocular. ***Ostiole*** 42–44 μm diam., central. ***Peridium*** 35–40 μm wide, thick-walled, outer layers composed of 3–5 layers dark brown cells of ***textura angularis***, becoming hyaline towards the inner region. ***Pseudoparaphyses*** 3–4.5 μm wide, hyaline, frequently aseptate. ***Asci*** 63.5–77 × 20–24.5 μm (*x̄* = 70 × 22.5 μm, n = 30), short stipe, clavate, thick-walled, bitunicate, 8-spored, irregularly biseriate. ***Ascospores*** 22.5–25 × 9.5–11 μm (*x̄* = 24 × 10 μm, n = 30), L/W ratio = 2.6, oblong, ovate to sub-clavate, (0–)1-septate, slightly constricted at the septum, hyaline to dark brown, moderately thick-walled, straight or inequilateral, basal cell tapering towards the acute end. **Asexual morph**: Not observed.

**Figure 14.**
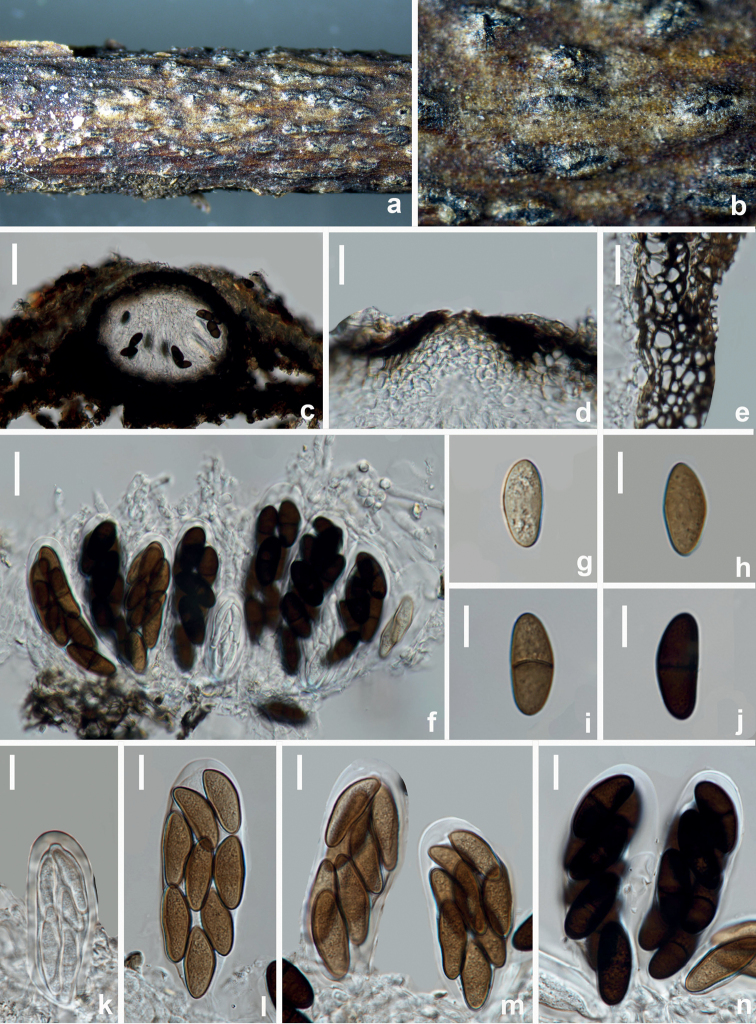
*Dothiorellazanthoxyli* (HKAS 125893, holotype) **a, b** appearance of ascomata on natural substrate **c** vertical section of ascoma **d** ostiole **e** section of peridium **f, k–n** asci **g–j** brown ascospores. Scale bars: 50 μm (**c**); 20 μm (**d–f**); 10 μm (**g–n**).

##### Culture characteristics.

Ascospores germinate on PDA within 12 h. Colonies growing on PDA, reaching a diameter of 4 cm after five days at 25 °C, effuse, velvety, with entire to slightly undulate edge. Surface initially white and later turning dark olivaceous from the surrounding of the colony. Dark gray in reverse.

##### Materials examined.

China, Sichuan Province, Yanan City, Hanyuan County, 29°16'51"N, 102°37'48"E, elevation 1,689 m, on dead branches of *Zanthoxylumbungeanum* (Rutaceae), 30^th^ October 2021, W.L Li, 504 (HKAS 125893, holotype), ex-type living culture UESTCC 22.0082 = CGMCC 3.24159; *ibid*., 506 (HUEST 22.0084), living culture UESTCC 22.0083; *ibid*., 507 (HUEST 22.0085), living culture UESTCC 22.0084. Additional sequences: LSU: OQ164839 (CGMCC 3.24159), OQ164840 (UESTCC 22.0083), OQ164841 (UESTCC 22.0084).

##### Notes.

Three strains of *Dothiorellazanthoxyli* isolated from *Zanthoxylumbungeanum* correspond well with sexual morph of *Dothiorella* described by Phillips et al. (2013), but morphologically differ from other species (*Do.camelliae*, *Do.iberica* and *Do.sarmentorum*) in the size of ascomata and asci (Table 3). A comparison of ITS and *tef1* nucleotides shows that *Do.zanthoxyli* is significantly different from its sister species, *Do.camelliae* by 4/550 bp (0.72%) in ITS and 14/242 bp (5.8%) in *tef1*. In the phylogenetic analysis, these two species formed two distinct clades in *Dothiorella* (Fig. 4).

#### ﻿ Neofusicoccum parvum

Taxon classificationFungiBotryosphaerialesBotryosphaeriaceae

(Pennycook & Samuels) Crous, Slippers & A.J.L. Phillips, Stud. Mycol. 55: 248. (2006).

BFC77A5D-1038-599E-B768-6F7FE5DFF798

 500879

Fig. 15

Fusicoccum parvum Pennycook & Samuels, Mycotaxon 24: 455. 1985. Basionym.
= Botryosphaeriaparva Pennycook & Samuels, Mycotaxon 24: 455. 1985.

##### Description.

*Saprobic* on decaying branches of *Idesiapolycarpa*. **Sexual morph: *Ascomata*** 284–321 × 129–223 μm (*x̄* = 302.5 × 176 μm, n = 10), pseudothecial, forming a botryose aggregation of up to 30, solitary or gregarious, stromatic, immersed, partially erumpent when mature, dark brown to black, more or less circular, multiloculate, individual locules 143.5–161 μm diam, thick-walled. ***Peridium*** 59–78 μm diam., composed of several layers of thick-walled, pale brown cells of ***textura angularis*. *Ostiole*** 43.5–58 μm wide, circular, central, papillate. ***Asci*** 95–99 × 20–21.5 μm (*x̄* = 97 × 20.5 μm, n = 30), (6–)8‐spored, bitunicate, fissitunicate, cylindrical to clavate, apex rounded with an ocular chamber, sometimes short pedicellate. ***Ascospores*** 18.5–23 × 7–10.5 μm (*x̄* = 20.5 × 9 μm, n = 30), L/W ratio = 3, fusoid to ovoid, with tapered ends and appearing spindle-shape, hyaline, aseptate, externally smooth, internally finely verruculose, biseriate in ascus. **Asexual morph**: Not observed.

**Figure 15.**
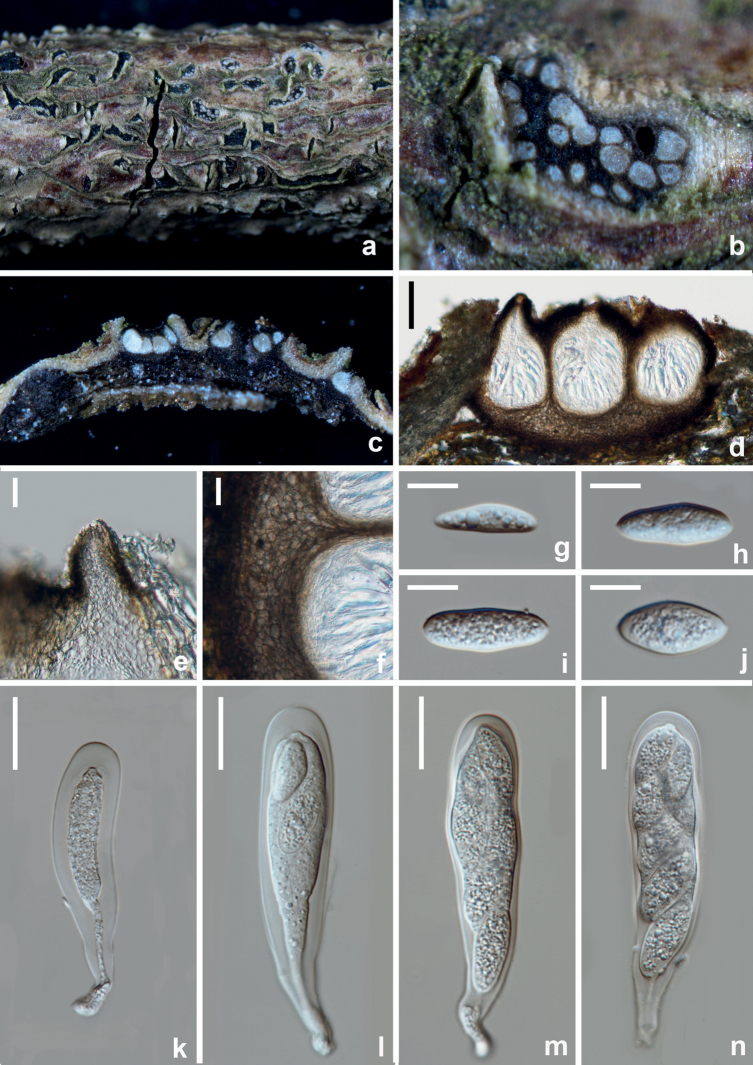
*Neofusicoccumparvum* (HUEST 22.0097, new host record) **a, b** appearance of ascomata on natural substrate **c, d** vertical section of ascomata **e** ostiole **f** section of peridium **g–j** ascospores **k, l** immature asci **m, n** mature asci. Scale bars: 100 μm (**d**); 25 μm (**e, f**); 10 μm (**g–j**); 20 μm (**k–n**).

##### Culture characteristics.

Ascospores germinate on PDA within 12 h. Colonies growing on PDA, reaching a diam., of 7 cm after five days at 25 °C, effuse, velvety, with entire to slightly undulate edge. Surface initially white and later turning dark olivaceous from the surrounding of the colony and dark gray in reverse.

##### Materials examined.

China, Sichuan Province, Leshan City, Jingyan County, 29°30'27"N, 103°57'14"E, elevation 682 m, on dead branches of *Idesiapolycarpa*, 23^th^ July 2021, W.L Li, STZ 327 (HUEST 22.0095), living culture UESTCC 22.0094; *ibid*., STZ 359 (HUEST 22.0094), living culture UESTCC 22.0093; *ibid*., Leshan City, Shizhong Distinct, 29°42'13"N, 103°52'25"E, elevation 356 m, on dead branches of *Paeoniasuffruticosa*, 23^th^ July 2021, W.L Li, YMD 366 (HUEST 22.0096), living culture UESTCC 22.0095; *ibid*., Guangyuan City, Qingchuan County, 32°40'38"N, 105°28'57"E, elevation 638 m, on dead branches of *Verniciafordii*, 20^th^ April 2021, W.L. Li, YT 175 (HUEST 22.0097), living culture UESTCC 22.0096.

##### Notes.

The morphology of our collections obtained from decaying woody oil plants are similar to the original description of *Neofusicoccumparvum* (Crous et al. 2006). In the multi-gene phylogenetic analysis, these four isolates clustered together (ML/BI 75%/0.99) with the ex-type of *N.parvum*. *Neofusicoccumparvum* has a wide range of hosts and has a worldwide distribution (Phillips et al. 2013). This is the first report of *N.parvum* on *Idesiapolycarpa*.

#### ﻿ Sardiniella guizhouensis

Taxon classificationFungiBotryosphaerialesBotryosphaeriaceae

Y.Y. Chen & Jian K. Liu. Phytotaxa 508 (2): 190. (2021).

314068C3-AE3A-530F-91D6-43919A774BC5

 558352

Fig. 16

##### Description.

*Saprobic* on decaying branches of *Pistaciachinensis*. **Sexual morph**: Not observed. **Asexual morph: *Conidiomata*** 223–232 × 150–176 μm (*x̄* = 227.5 × 163 μm, n = 10), dark brown to black, globose, submerged in the substrate, partially erumpent at maturity, ostiolate. ***Ostiole*** 28.5–45 μm diam., circular, central. *Peridium* 21–30 μm thick, composed of dark brown thick-walled cells of ***textura angularis***, becoming thin-walled and hyaline towards the inner region. ***Conidiophores*** reduced to conidiogenous cells. ***Conidiogenous cells*** 6–9.5 × 3.5–5 μm (*x̄* = 7.5 × 4 μm, n = 20), hyaline, short obpyriform to subcylindrical, holoblastic, indeterminate. ***Conidia*** 20.5–24 × 11.5–14 μm (*x̄* = 22 × 13 μm, n = 30), L/W ratio = 1.6, ellipsoid to ovoid with both ends rounded, hyaline, aseptate, externally smooth, internally finely verruculose.

**Figure 16.**
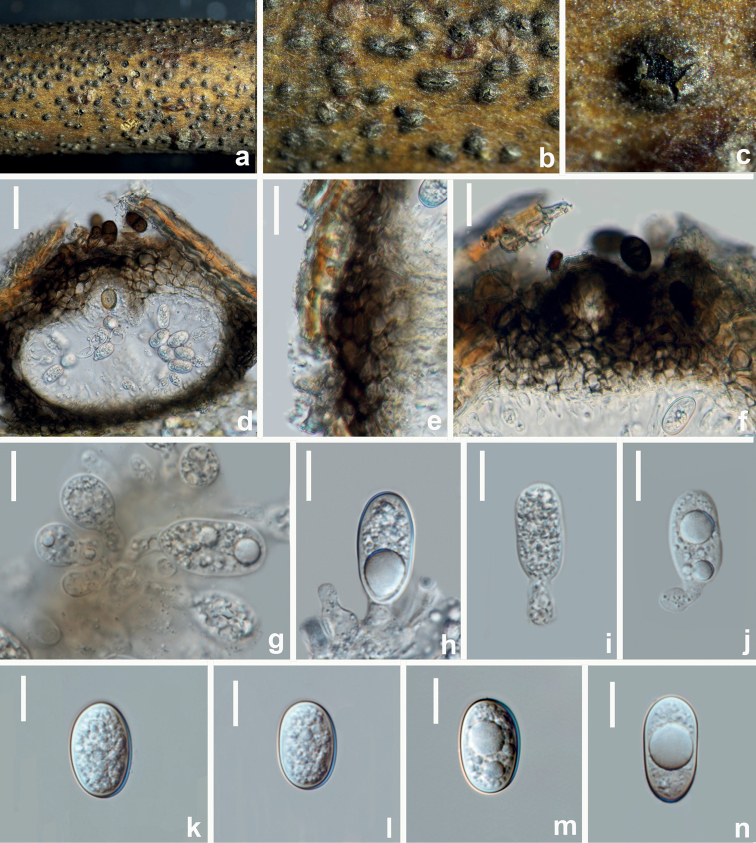
*Sardiniellaguizhouensis* (HUEST 22.0100, new host record) **a–c** appearance of conidiomata on natural substrate **d** vertical section of conidioma **e** section of peridium **f** ostiole **g–j** conidiogenous cells and developing conidia **k–n** conidia. Scale bars: 40 μm (**d**); 20 μm (**e, f**); 10 μm (**g–n**).

##### Culture characteristics.

Conidia germinate on PDA within 12 h. Colonies growing on PDA, reaching a diameter of 7 cm after five days at 25 °C, effuse, velvety, with entire to slightly undulate edge. Surface initially white and later turning dark olivaceous from the surrounding of the colony and dark gray in reverse.

##### Material examined.

China, Sichuan Province, Chengdu City, Pidu District, 29°16'50.70"N, 102°37'47.53"E, elevation 442 m, on dead branches of *Pistaciachinensis*, 19^th^ March 2021, W.L Li, 047 (HUEST 22.0101), living culture UESTCC 22.0100; *ibid*., 070 (HUEST 22.0102), living culture UESTCC 22.0101; *ibid*., 071 (HUEST 22.0100), living culture UESTCC 22.0099; *ibid*., 150 (HUEST 22.0098), living culture UESTCC 22.0097; *ibid*., 151 (HUEST 22.0099), living culture UESTCC 22.0098; *ibid*., A39 (HUEST 22.0103), living culture UESTCC 22.0102; *ibid*., A40 (HUEST 22.0104), living culture UESTCC 22.0103. Additional sequences: LSU: OQ164842 (UESTCC 22.0100), OQ164843 (UESTCC 22.0101), OQ164844 (UESTCC 22.0099), OQ164845 (UESTCC 22.0097), OQ164846 (UESTCC 22.0098), OQ164847 (UESTCC 22.0102).

##### Notes.

Seven isolates of our collection are morphologically similar to the original description of *Sardiniellaguizhouensis* (Chen et al. 2021). The multi-gene phylogenetic analysis showed that the newly obtained isolates clustered together with ex-type of *Sa.guizhouensis* (CGMCC 3.19222) and this is the first report of *Sa.guizhouensis* from *Pistaciachinensis*.

#### ﻿ Sphaeropsis citrigena

Taxon classificationFungiBotryosphaerialesBotryosphaeriaceae

(A.J.L. Phillips, P.R. Johnst. & Pennycook) A.J.L. Phillips & A. Alves. Stud. Mycol. 76, 157. (2013).

5A171D09-0825-5B7D-9CB2-39A01C0391C2

 805463

Fig. 17

##### Description.

*Saprobic* on decaying branches of *Camelliaoleifera*. **Sexual morph: *Ascomata*** 219–252 × 216–241 μm (*x̄* = 235.5 × 228.5 μm, n = 10), brown to black, solitary or aggregated, immersed, becoming erumpent, ostiolate. ***Ostiole*** 71–92 μm, central, relatively broad. ***Peridium*** 37.5–45 μm diam., composed of several layers of dark brown cells of ***textura angularis*. *Pseudoparaphyses*** 1.5–2 μm wide, hyaline, smooth, septate. ***Asci*** 93.5–107 × 28.5–33 μm (*x̄* = 100 × 30.5 μm, n = 30), bitunicate, 8-spored, stipitate, thick-walled, with well-developed apical chamber. ***Ascospores*** 29–35 × 13–15 μm (*x̄* = 32 × 14 μm, n = 30), L/W ratio = 2.3, yellowish brown to dark brown, ellipsoid to ovoid with both ends rounded, with an apiculus at either end, aseptate, externally smooth, internally finely verruculose, widest in middle to upper third. **Asexual morph**: Not observed.

**Figure 17.**
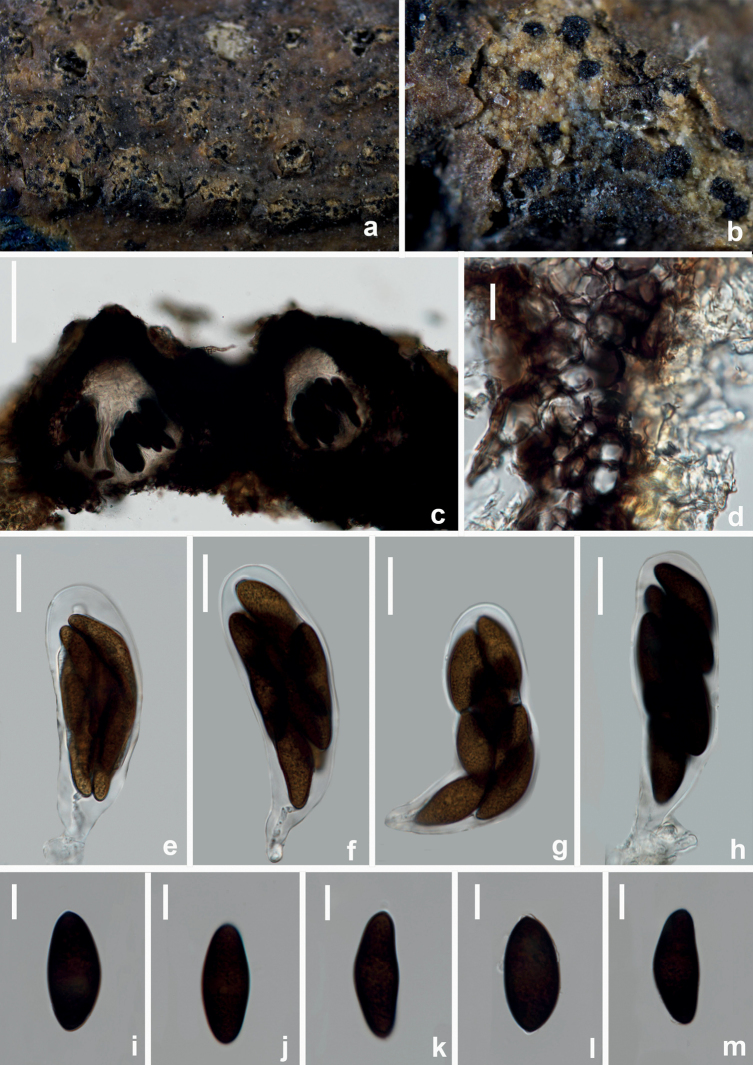
*Sphaeropsiscitrigena* (HUEST 22.0107, new host record) **a, b** appearance of ascomata on natural substrate **c** vertical section of ascomata **d** section of peridium **e–h** mature asci **i–m** dark brown ascospores. Scale bars: 100 μm (**c**); 20 μm (**d–h**); 10 μm (**i–m**).

##### Culture characteristics.

Ascospores germinate on PDA within 12 h. Colonies growing on PDA, reaching a diam. of 7 cm after five days at 25 °C, effuse, velvety, with entire to slightly undulate edge. Surface initially white and later turning dark olivaceous from the surrounding of the colony and dark gray in reverse.

##### Materials examined.

China, Sichuan Province, Chengdu City, Pidu District, 31°54'10"N, 104°55'57"E, 656 m, on dead branches of *Camelliaoleifera*, 10^th^ June 2021, W.L Li, 285 (HUEST 22.0107), living culture UESTCC 22.0106; *ibid*., on dead branches of *Acertruncatum*, 30°19'57"N, 103°59'47"E, elevation 442 m, 19^th^ March 2021, W.L Li, A33 (HUEST 22.0106), living culture UESTCC 22.0105. Additional sequence: LSU: OQ164848 (UESTCC 22.0105).

##### Notes.

The phylogenetic tree shows that two isolates of *Sphaeropsis* from our collection clustered together with the ex-type strain of Sp. *citrigena* (ICMP 16812) with high bootstrap support (ML/BI 100%/1). *Sphaeropsiscitrigena* was first described as *Phaeobotryosphaeriacitrigena* by Phillips et al. (2008), later transferred to *Sphaeropsis* based on morphological and phylogenetic analyses (Phillips et al. 2013). The new collection (UESTCC 22.0105) isolated from *Camelliaoleifera* resembles Sp. *citrigena* isolated from *Citrussinensis* (Phillips et al. 2013) in the shape of asci and ascospores, though their asci are somewhat smaller than those of Sp. *citrigena* (93.5–107 × 28.5–33 μm vs. 180–230 × 35–43 μm). In addition, there are no base pair differences in ITS and *tef1* sequences of these two strains. We, thus, identify the new collection as Sp. *citrigena* and this is the first record of Sp. *citrigena* from *Camelliaoleifera*.

#### ﻿ Sphaeropsis guizhouensis

Taxon classificationFungiBotryosphaerialesBotryosphaeriaceae

Y.Y. Chen, A. J. Dissanayake & Jian K. Liu., J. Fungi 7, 893. (2021).

4961A8D8-D572-55FF-AD9C-9B9274C6A454

 558475

Fig. 18

##### Description.

*Saprobic* on decayed branched of *Camelliaoleifera*. **Sexual morph: *Ascostromata*** 166–198 × 146.5–175 μm (*x̄* = 182 × 160.5 μm, n = 20), initially immersed under host epidermis, becoming semi‐immersed to erumpent, solitary or gregarious, uniloculate, black, globose to subglobose, membraneous, ostiolate. ***Ostiole*** 75–80 μm wide, central, papillate, pale brown, relatively broad, periphysate. ***Peridium*** 23–27 μm wide, comprising 3–5 layers of relatively thick‐walled, dark brown to black‐walled cells arranged in a ***textura angularis*. *Pseudoparaphyses*** 2–2.5 μm diam., hyphae‐like, numerous, embedded in a gelatinous matrix. ***Asci*** 87.5–135 × 28.5–35 μm (*x̄* = 111 × 32 μm, n = 20), 8‐spored, bitunicate, fissitunicate, cylindrical to clavate, sometimes short pedicellate, mostly long pedicellate, apex rounded with an ocular chamber. ***Ascospores*** 28.5–33 × 13–15 μm (*x̄* = 30.5 × 14 μm, n = 20), overlapping uniseriate to biseriate, ellipsoidal to obovoid, pale brown to dark brown, septate, slightly wide at the center, minutely guttulate, smooth‐walled. **Asexual morph**: Not observed.

**Figure 18.**
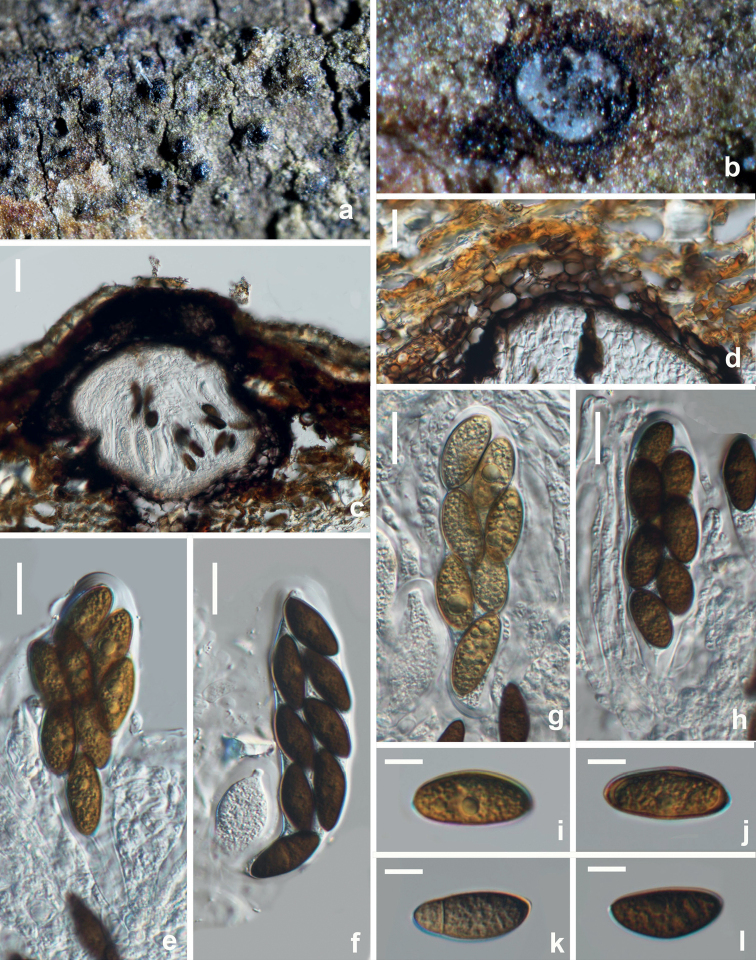
*Sphaeropsisguizhouensis* (HUEST 22.0105, new host record) **a, b** appearance of ascomata on natural substrate **c** vertical section of ascoma **d** section of peridium **e–h** mature asci **i–l** brown ascospores. Scale bars: 20 μm (**c–h**); 5 μm (**i–l**).

##### Culture characteristics.

Ascopores germinate on PDA within 12 h. Colonies growing on PDA, reaching a diam. of 7 cm after five days at 25 °C, effuse, velvety, with entire to slightly undulate edge. Surface initially white and later turning dark olivaceous from the surrounding of the colony and dark gray in reverse.

##### Material examined.

China, Sichuan Province, Chengdu City, Pidu District, on dead branches of *Pistaciachinensis*, 30°19'57"N, 103°59'47"E, elevation 442 m, 24^th^ March 2021, W.L Li, 290 (HUEST 22.0105), living culture UESTCC 22.0104.

##### Notes.

*Sphaeropsisguizhouensis* was introduced by Dissanayake et al. (2021) and isolated from an unknown host. One isolate obtained in the present study clustered with the ex-type isolate of *Sp.guizhouensis* (CGMCC 3.20352) in the phylogenetic analyses of combined ITS and *tef1* sequence data with high bootstrap support. A comparison of ITS and *tef1* shows that there are no base pair differences between the isolates of UESTCC 22.0104 and CGMCC 3.20352. The new collection is morphologically similar to *Sp.guizhouensis*, with immersed to erumpent, black ascostromata and biseriate, aseptate, ellipsoid to obovoid, thick‐walled conidia. In addition, ascospores become brown and septate when aged. Considering similar morphology and strong molecular evidence, we identify UESTCC 22.0104 as *Sp.guizhouensis* and this is the first record of *Sp.guizhouensis* on *Camelliaoleifera*.

### ﻿Diversity of Botryosphaerialean fungi collected in this study

**Figure 19.**
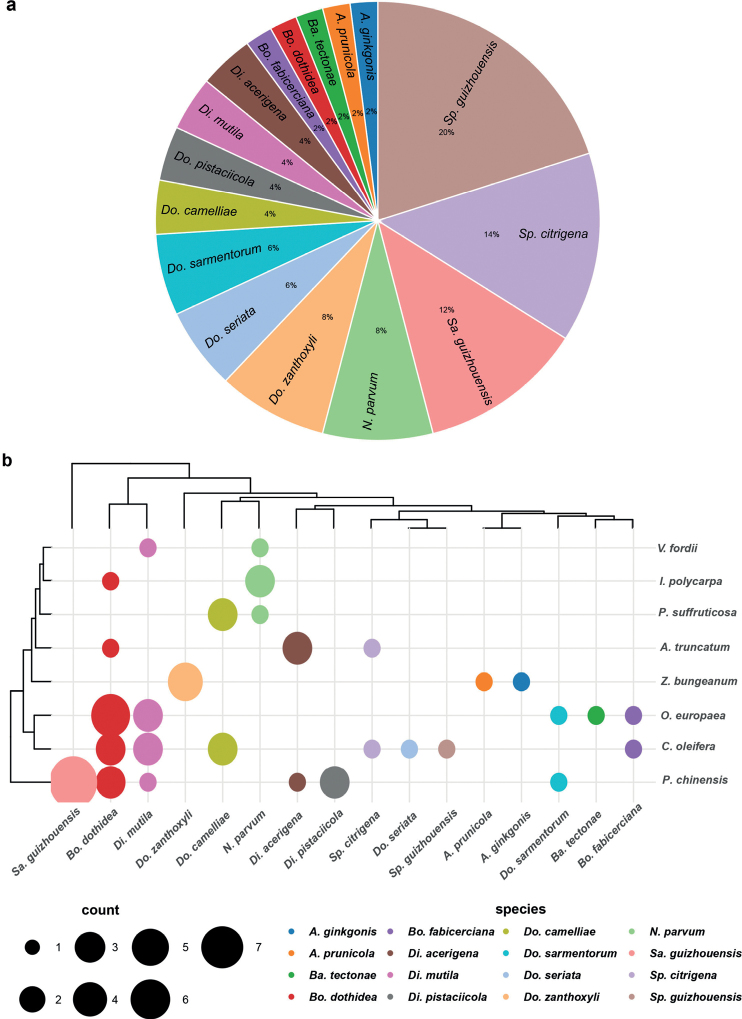
Botryosphaeriales species composition **a** the proportion of each species to the total number of isolates **b** the number of Botryosphaeriales fungi on each host and host distribution of species.

Based on the phylogenetic and morphological analyses, 50 Botryosphaeriales isolates collected from the five regions (Chengdu, Guangyuan, Leshan, Mianyang and Yaan City) in Sichuan Province were identified as 16 species. Of these, *Botryosphaeriadothidea* was the most prevalent species (20%), followed by *Sphaeropsisguizhouensis* (14%) and *Diplodiamutila* (12%) (Fig. 19a). *Aplosporellaginkgonis*, *Barriopsistectonae* and *Sphaeropsisguizhouensis* were identified only once. There are 14 isolates (28%) isolated from *Pistaciachinensis*, including *Di.acerigena*, *Di.mutila*, *Di.pistaciicola*, *Dothiorellasarmentorum* and *Sardiniellaguizhouensis*. Ten isolates were from *Camelliaoleifera* (20%), including *Bo.dothidea*, *Bo.fabicerciana*, *Di.mutila*, *Do.camelliae*, *Sp.citrigena* and *Sp.guizhouensis*. Nine isolates were from *Oleaeuropaea* (18%), including *Ba.tectonae*, *Bo.dothidea*, *Bo.fabicerciana*, *Di.mutila* and *Do.sarmentorum*. Relatively few strains were found on *Idesiapolycarpa*, *Paeoniasuffruticosa* and *Verniciafordii*, as each host presents two species, respectively. As of final conclusion, *Bo.dothidea* were isolated from five hosts, *Di.mutila* were isolated from four hosts, *N.parvum* were isolated from three hosts, *Bo.fabicerciana*, *Di.acericola*, *Do.camelliae*, *Do.sarmentorum* and *Sp.citrigena* were isolated from two hosts, but several fungal isolates were only isolated from one host species, such as *A.prunicola*, *Sa.guizhouensis* and *Sp.guizhouensis* (Fig. 19b).

## ﻿Discussion

In this study, 48 Botryosphaeriaceae isolates and two Aplosporellaceae isolates were obtained from woody oil plants in Sichuan Province, China, and they were identified as 16 species based on morphological characters and multi-gene phylogenetic analyses. These species included *Aplosporellaprunicola*, *A.ginkgonis*, *Barriopsistectonae*, *Botryosphaeriadothidea*, *Bo.fabicerciana*, *Diplodiaacerigena*, *Di.mutila*, *Di.pistaciicola*, *Di.seriata*, *Dothiorellacamelliae*, *Do.sarmentorum*, *Do.zanthoxyli*, *Neofusicoccumparvum*, *Sardiniellaguizhouensis*, *Sphaeropsiscitrigena* and *Sp.guizhouensis*. Of these, *Di.acerigena*, *Di.pistaciicola*, *Do.camelliae* and *Do.zanthoxyli* are introduced as novel species. Descriptions, illustrations and notes were provided for 13 species, and only sequences data were provided for the remaining three species viz. *Barriopsistectonae*, *Botryosphaeriadothidea* and *Bo.fabicerciana* due to low specimen quality.

According to previous studies, *Barriopsistectonae*, *Sardiniellaguizhouensis*, *Sphaeropsiscitrigena* and *Sp.guizhouensis* have limited geographical distribution. So far, *Barriopsistectonae* has been reported from China, Thailand and South Africa (Doilom et al. 2014; Dissanayake et al. 2021). *Sardiniellaguizhouensis* and *Sphaeropsisguizhouensis* were only found in China while *Sp.citrigena* was isolated from China, Colombia and New Zealand. It’s worth noting that most of the species obtained from this study were also reported previously from Guizhou province (Dissanayake et al. 2021). Earlier studies have shown that the distribution of Botryosphaeriaceae species is influenced by the climate condition (Úrbez-Torres et al. 2006; Pitt et al. 2010; Li et al. 2020; Vivas et al. 2021). Thus, we speculate that the adjacent geographical location and similar climatic conditions may be important reasons for the similarity of fungal species isolated from the Sichuan and Guizhou provinces.

The remaining Botryosphaeriaceae species identified in this study are all well-known and reported from various geographic regions. *Botryosphaeriadothidea*, *Di.seriata* and *Ne.parvum* are recognized to be globally distributed while *Di.mutila* and *Do.sarmentorum* are founded only in the temperate and Mediterranean areas. In addition, these species have a broad host range. Batista et al. (2021) reported *Neofusicoccumparvum* from 223 hosts, *B.dothidea* from 403 hosts and *Di.seriata* from 121 hosts. *Diplodiamutila* and *Di.seriata* have previously been reported on *Oleaeuropaea* in Uruguay (Hernández-Rodríguez et al. 2022). *Botryosphaeriadothidea* was recently isolated from diseased *Camelliaoleifera* in China (Hao et al. 2022). In this study, *Bo.dothidea*, *Di.mutila* and *Ne.parvum* occurred on most of the woody oil plants species we examined. However, some common genera e. g. *Lasiodiplodia*, *Neodeightonia* and *Phaeobotryon* have never been collected from this group of hosts (Fig. 19). The absence of these genera from there is likely a sampling effect.

*Aplosporella* (Aplosporellaceae) was introduced by Spegazzini (1880) with *A.chlorostroma* as the genetic type. In a previous study, *Aplosporella* represents anamorph lineage within the Botryosphaeriaceae. Slippers et al. (2013) later proposed the family Aplosporellaceae to accommodate *Aplosporella* and *Bagnisiella*. *Aplosporella* species are infrequently isolated in China. *Aplosporellaginkgonis*, isolated from Gansu Province, was first descripted by Du et al. (2017) while *Aplosporellamacropycnidia* was reported in Yunnan Province. Subsequently, Jiang et al. (2021) isolated a new collection of *A.prunicola*. However, other species have not been recorded in China. Our study revealed new host records of *A.ginkgonis* and *A.prunicola*. Though the phylogenetic analyses indicated that *A.yalgorensis* and *A.prunicola* have a low genetic divergence (Taylor et al. 2008, in this study), *A.yalgorensis* is still considered as a different species as it differs from other *Aplosporella* species (including *A.prunicola*) by its pitted conidial walls.

Though there are more than 1,000 *Diplodia* epithets listed in Index fungorum (www. Index Fungorum. Accessed in November 2022), presently only 30 species are accepted in this genus based on phylogenetic analyses (Slippers et al. 2017; Wu et al. 2021). Holomorphic species in *Diplodia* are *Di.tsugae*, *Di.seriata*, *Di.mutila* and *Di.sapinea*. This study revealed two previously known *Diplodia* species, *Di.mutila* and *Di.seriata*, and two new species, *Di.acerigena* and *Di.pistaciicola*. Among them, *Di.acerigena* is a holomorphic species, as its sexual stage was observed on the dead branches of *Acertruncatum*, and the asexual stage produced on culture (PDA). However, the sexual morph of *Di.mutila* and *Di.pistaciicola*, as well as the asexual morph of *Di.seriata* have not been observed on woody oil plants.

*Dothiorella* was established by Saccardo with *Do.pyrenophora* as the type species (Saccardo 1880). Recently, *Dothiorella* encountered a series of revisions as many species in this genus have been reduced to synonymy, such as *Do.americana*, *Do.eriobotryae* and *Do.iberica* (Dissanayake et al. 2021; Zhang et al. 2021). So far, 31 species are valid in *Dothiorella*. Most of the species were reported as the asexual morph of *Dothiorella* and the sexual stage is rarely founded on nature (Dissanayake et al. 2016). Phillips et al. (2013) initiated a link of asexual-sexual morph for *Do.sarmentorum*, *Do.iberica* and *Do.vidmadera*. However, the latter two species were synonymized under *Do.sarmentorum* (Zhang et al. 2021). In this study, two new species *Do.camelliae* and *Do.zanthoxyli* are introduced based on their sexual morphs as well as strong molecular evidences. Besides, new collections of *Do.sarmentorum* is reported on *Pistaciachinensis* for the first time.

Multiple molecular systematic studies, mainly of pathogenic fungi of woody plants (Phillips et al. 2013; Slippers et al. 2013; Dissanayake et al. 2021; Zhang et al. 2021), have generated a robust phylogeny for Botryosphaeriaceae. However, the classification and identification of some species in this family remains a major challenge, due to the reasons 1) With the increase of the number of Botryosphaeriaceae species, morphological feature of inter-genera and inter-species is vague, 2) Some species occurred as asexual morph on nature and it is difficult to establish the link of asexual and sexual morph, 3) In general, Botryosphaeriaceae species do not show an obvious host specialization, while some populations displayed a certain degree of host association. Thus, the traditional host-based classification system made taxonomic position confusion of some species. Therefore, collection of more fresh specimens is very important for better understanding the life cycle of Botryosphaeriaceae species, their host range (e. g. native plants) and potential pathogenicity.

## Supplementary Material

XML Treatment for Aplosporella ginkgonis

XML Treatment for Aplosporella prunicola

XML Treatment for Diplodia acerigena

XML Treatment for Diplodia mutila

XML Treatment for Diplodia pistaciicola

XML Treatment for Diplodia seriata

XML Treatment for Dothiorella camelliae

XML Treatment for Dothiorella sarmentorum

XML Treatment for Dothiorella zanthoxyli

XML Treatment for Neofusicoccum parvum

XML Treatment for Sardiniella guizhouensis

XML Treatment for Sphaeropsis citrigena

XML Treatment for Sphaeropsis guizhouensis

## Supplementary materials

Supplementary material 1 Taxa and GenBank accession numbers of sequences used in this study Data type table (excel document) https://binary.pensoft.net/file/853842This dataset is made available under the Open Database License (http://opendatacommons.org/licenses/odbl/1.0/). The Open Database License (ODbL) is a license agreement intended to allow users to freely share, modify, and use this Dataset while maintaining this same freedom for others, provided that the original source and author(s) are credited.Wen-Li Li, Rui-Ru Liang, Asha Dissanayake, Jian-Kui Liu
